# Neuroinflammatory responses and blood–brain barrier injury in chronic alcohol exposure: role of purinergic P2 × 7 Receptor signaling

**DOI:** 10.1186/s12974-024-03230-4

**Published:** 2024-09-28

**Authors:** Namdev S. Togre, Naveen Mekala, Priyanka S. Bhoj, Nikhita Mogadala, Malika Winfield, Jayshil Trivedi, Deborah Grove, Sudhir Kotnala, Slava Rom, Uma Sriram, Yuri Persidsky

**Affiliations:** Department of Pathology and Laboratory Medicine, Lewis Katz School of Medicine, Temple University, Philadelphia, PA 19140 USA

**Keywords:** CIE, Blood-brain barrier, ATP, P2 × 7R, Extracellular vesicles

## Abstract

Alcohol consumption leads to neuroinflammation and blood‒brain barrier (BBB) damage, resulting in neurological impairment. We previously demonstrated that ethanol-induced disruption of barrier function in human brain endothelial cells was associated with mitochondrial injury, increased ATP and extracellular vesicle (EV) release, and purinergic receptor P2 × 7R activation. Therefore, we aimed to evaluate the effect of P2 × 7R blockade on peripheral and neuro-inflammation in ethanol-exposed mice. In a chronic intermittent ethanol (CIE)-exposed mouse model, P2 × 7R was inhibited by two different methods: Brilliant Blue G (BBG) or gene knockout. We assessed blood ethanol concentration (BEC), brain microvessel gene expression by using RT2 PCR array, plasma P2 × 7R and P-gp, serum ATP, EV-ATP, number of EVs, and EV mtDNA copy numbers. An RT2 PCR array of brain microvessels revealed significant upregulation of proinflammatory genes involved in apoptosis, vasodilation, and platelet activation in CIE-exposed wild-type animals, which were decreased 15–50-fold in BBG-treated–CIE-exposed animals. Plasma P-gp levels and serum P2 × 7R shedding were significantly increased in CIE-exposed animals. Pharmacological or genetic suppression of P2 × 7R decreased receptor shedding to levels equivalent to those in control group. The increase in EV number and EV-ATP content in the CIE-exposed mice was significantly reduced by P2 × 7R inhibition. CIE mice showed augmented EV-mtDNA copy numbers which were reduced in EVs after P2 × 7R inhibition or receptor knockout. These observations suggested that P2 × 7R signaling plays a critical role in ethanol-induced brain injury. Increased extracellular ATP, EV-ATP, EV numbers, and EV-mtDNA copy numbers highlight a new mechanism of brain injury during alcohol exposure via P2 × 7R and biomarkers of such damage. In this study, for the first time, we report the in vivo involvement of P2 × 7R signaling in CIE-induced brain injury.

**Supplementary Information:**

The online version contains supplementary material available at 10.1186/s12974-024-03230-4.

## Introduction

Alcohol abuse and its detrimental effects on the central nervous system (CNS) have long been recognized as significant public health concerns. Excessive alcohol consumption is listed as one of the leading risk factors for population health, disease, disability, and death worldwide. According to the CDC’s Morbidity and Mortality Weekly Report (MMWR), an average annual number of deaths increased up to 29% beginning from 2016 to 2021 [1]. A recent report, “World Alcohol and Health Situation,” from the World Health Organization indicated that more than 3 million deaths attributed to alcohol consumption correspond to one death every 10 s.

Several studies have reported that excessive alcohol consumption causes damage to various organs [2]. The key mechanisms underlying alcohol-induced neurotoxicity involve neuroinflammation and blood‒brain barrier (BBB) disruption, which contribute to neuronal damage and dysfunction [3, 4]. Loss of BBB function is associated with increased permeability and reduced expression of key proteins in the BBB [5, 6]. Disruption of BBB integrity leads to the infiltration of peripheral immune cells, cytokine release, and subsequent neuroinflammatory responses, exacerbating neuronal injury [7, 8].

Purinergic receptor-mediated signaling is essential in the CNS for maintaining physiological neural cell function and has emerged as a crucial modulator of neuroinflammation and BBB function [9, 10]. Among purinergic receptors, the P2 × 7R is involved in inflammatory processes and cell death cascades [11, 12]. Additionally, its association with BBB disruption is of interest [13]. Adverse cellular conditions, such as stress and cellular damage, lead to an increase in extracellular ATP (eATP) concentrations, which act as damage-associated molecular patterns (DAMPs); supraphysiologic ATP concentrations are responsible for P2 × 7R activation. In vitro chronic alcohol exposure of human macrophages results in the activation of the P2 × 7R-mediated Nod-like receptor pyrin domain containing 3 (NLRP3) inflammasome pathway, which causes the secretion of interleukin 1 beta (IL-1β) [14]. Moreover, ethanol (EtOH)-dependent P2 × 7R overactivation causes alcohol-induced BBB damage with increased levels of proinflammatory cytokines IL-1β, tumor necrosis factor alpha (TNF-α), and interleukin-6 (IL-6) in mice [15]. In a series of investigations, our laboratory revealed the effects of EtOH exposure on brain microvascular endothelial cells (BMVECs) and demonstrated a compelling link between substance exposure and dysregulation of purinergic signaling pathways. EtOH-exposed BMVECs showed the mito-stress and enhanced eATP release, which were blocked by P2 × 7R antagonist [16–18].

Extracellular ATP stimulates P2 × 7R, which triggers extracellular vesicle (EV) shedding [19, 20]. EVs are cargo-carrying cell-derived vesicles which can communicate between originating and recipient cells. Several reports have stated the changes in cargo composition based on host cell health status [21, 22]. P2 × 7R activation can change EV proteome and may be involved in the propagation of inflammation. EVs carry cytokines, various mRNAs, lipids, and ATP molecules [21, 23]. Chronic EtOH exposure increases levels of proinflammatory molecules in EVs [23–25]. Studies have also detected the presence of mitochondrial DNA (mtDNA) fragments with DAMP-like properties in EVs isolated after chronic EtOH exposure [25].

Several studies have reported the undeniable role of P2 × 7R signaling in BBB injury in vitro [11, 17, 26–29]. However, the precise mechanisms underlying P2 × 7R-mediated effects in alcohol-induced neuroinflammation in vivo remain incompletely understood. In earlier studies, investigators have tested P2 × 7R-inhibitory and neuroprotective effects of BBG in mouse models of Parkinson, Alzheimer and alcohol-induced steatohepatitis [30–33]. In this study, we hypothesize that blocking of P2 × 7R signaling either by administration of BBG (P2 × 7R blocker) or P2 × 7R genetic deletion (P2 × 7R^-/-^) will reduce neuroinflammation and BBB injury in chronic EtOH-exposed mice.

In this study, we found that pharmacologic or genetic inhibition of P2 × 7R significantly decreased the levels of upregulated brain proinflammatory cytokines, circulating P2 × 7R, serum ATP levels, EVs, EV-ATP, and EV-mtDNA fragments in a mouse model of chronic intermittent exposure (CIE) to EtOH. Furthermore, the genes involved in apoptosis, vasodilation, and platelet activation, which were significantly upregulated in the brain microvessels of alcohol-exposed mice, were reduced in CIE-exposed mice treated with the P2 × 7R inhibitor.

## Materials and methods

### Animals

P2 × 7R^−/−^ C57BL/6 (B6.129P2-*P2rx7*^*tm1Gab*^*/*J, stock no. 005576) mice were obtained from the Jackson Laboratories (Bar Harbor, ME), cross bred with wild-type C57BL/6 mice, and genotyped at our animal facility. Also, C57BL/6 wild-type mice were obtained from the Jackson Laboratories. Male mice, aged 16–17 weeks, were used for the experiments. To achieve statistical significance, 5–15 mice were used in each experimental group. In the pharmacologically P2 × 7R-inhibited cohort, we grouped wild-type mice into four groups: air control (*n* = 7), BBG-treated–CIE-unexposed (BBG; *n* = 5), CIE-exposed (CIE; *n* = 7), and BBG-treated–CIE-exposed (BBG-CIE; *n* = 8) group. For the P2 × 7R^−/−^ cohort, we grouped mice in to following groups: wild-type air control (*n* = 8), P2 × 7R^−/−^ CIE-unexposed (P2 × 7R^−/−^; *n* = 6), wild-type CIE-exposed (CIE; *n* = 15), and P2 × 7R^−/−^ CIE-exposed (P2 × 7R^−/−^-CIE; *n* = 15) group. The mice were housed five per cage with food and water available *ad libitum* (12-h light-dark cycle). All in vivo experiments were approved by the Temple University Institutional Animal Care and Use Committee in accordance with guidelines based on the National Institutes of Health (NIH) Guide for Care and Use of Laboratory Animals and the Animal Research: Reporting in Vivo Experiments (ARRIVE) guidelines (www.nc3rs.org.uk/arrive-guidelines; accessed on March 19, 2022).

### CIE and BBG injections

**Fig. 1:**
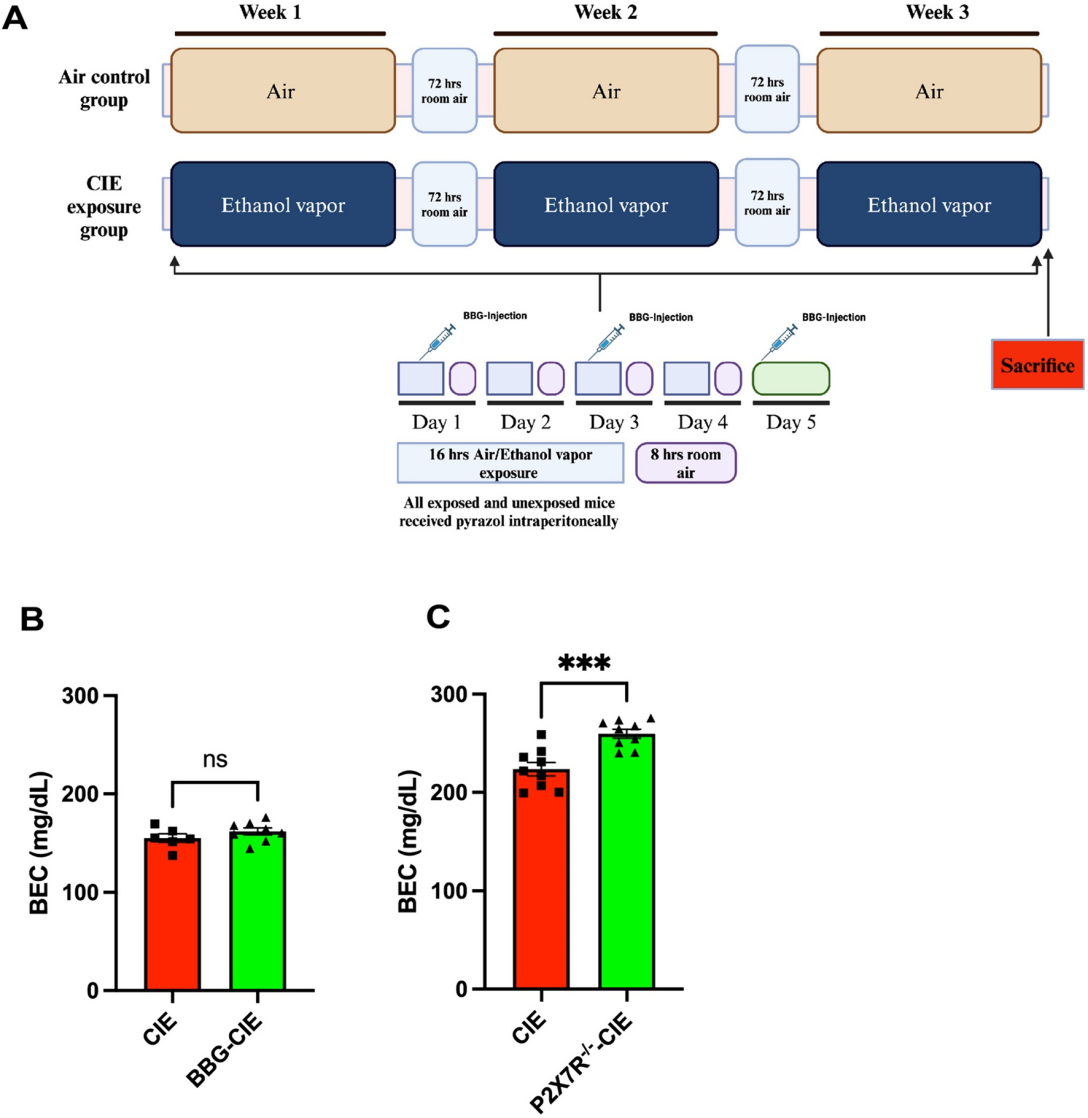
Schematic of the experiment and blood ethanol concentrations (BECs). **(A)** CIE exposure paradigm. Created with BioRender.com. **(B)** and **(C)** BECs were assessed to ensure that pathophysiologically relevant ethanol levels were obtained at the end of the experiment. The mean BECs were 154.98 ± 10.70 mg/dL, 161 ± 10.19 mg/dL, 223.59 ± 20.15 mg/dL, and 259.73 ± 13.73 mg/dL in the CIE-exposed and BBG-treated–CIE-exposed groups of the pharmacologically P2 × 7R-inhibited cohort **(B)** and wild-type CIE-exposed, and P2 × 7R^−/−^ CIE-exposed groups of the P2 × 7R^−/−^ cohort **(C)**, respectively. When compared with wild-type CIE-exposed group, the P2 × 7R^−/−^ CIE-exposed group showed significant difference in the BEC. A two-tailed t test was used for the statistical analyses. The values are presented as the mean ± SEM; *n* = 6–9; *** *p* < 0.0004 and ns = nonsignificant compared with the CIE-exposed mice

A mouse model of CIE exposure was developed as described previously [34–36] with the following modifications. All the mice in the CIE-exposed groups were exposed to continuous ethanol vapor for 16 h, followed by 8 h in room air each day for four days a week (1 cycle; Fig. 1A). The exposure cycle was repeated three times. Before placing the mice in ethanol vapor, an intraperitoneal (i.p.) injection of an alcohol dehydrogenase inhibitor, pyrazole (P56607-5G; Merck, USA, 85 mg/kg), and a loading dose of 1.0 g/kg ethanol (32801; Decon labs Inc.) (20% w/v) in 0.9% saline were given to initiate and maintain stable ethanol intoxication [37, 38]. All the mice in experimental groups were injected with 85 mg/kg pyrazole in saline [39, 40]. To deliver ethanol vapor, 190-proof ethanol was volatilized, mixed with fresh air at a rate of 10 L/min, and then pumped into the ethanol inhalation chamber. At the end of every cycle, a 2 mL air sample was drawn through a port in the chamber door to measure the amount of ethanol present in the chamber. The BBG was prepared according to the previously described procedure [41]. Before placement into exposure chamber, mice from the BBG-treated–CIE-unexposed and BBG-treated–CIE-exposed groups were injected i.p. with 45 mg/kg mouse body weight BBG ([ab120389; Abcam] in 100 µL of 0.9% saline) to inhibit P2 × 7R in vivo.

### Blood ethanol concentrations

At the end of the experiment, blood ethanol concentrations (BECs) were measured [39]. Blood samples were collected in 0.5 M EDTA (pH 8) through the submandibular vein punch immediately after removal of the mice from the ethanol vapor chamber. The serum samples were subjected to a spectrophotometric enzymatic assay (ECET-100TM Ethanol Assay Kit; BioAssay Systems, San Francisco, USA).

### Brain microvessel isolation

Mouse brain microvessels were isolated using an earlier published protocol with some modifications [18, 42, 43]. In brief, the mice were perfused with saline, and the brains were harvested. All the following steps were carried out on ice. Following a wash in PBS and removal of the cerebellum, meninges, and large superficial blood vessels, the right hemisphere of the brain was homogenized in 1 mL of ice-cold Hank’s balanced salt solution (HBSS) using a Dounce homogenizer (357538; Grienger, Philadelphia, USA) (0.25 mm clearance). Overall, the resulting homogenate was centrifuged at 1000 × *g* for 10 min, and the pellet was resuspended in ~ 5 mL of cold 17.5% dextran and centrifuged for 15 min at 4400 × *g* at 4 °C. Using a cut tip, the supernatant containing the myelin layer was removed, and the remaining pellet was resuspended in ~ 5 mL of HBSS containing 1% BSA. After the suspension was broken up using a 10 mL pipet in a Petri dish, it was passed through a 100 μm mesh nylon filter. The collected filtrate was passed through a 40 μm mesh nylon filter. The microvessels retained in the filter were collected by inverting the filter and rinsing it with 3 mL of HBSS containing 1% BSA. Finally, after centrifugation, the microvessels were collected and stored at -80 °C for further processing.

### Gene expression profiling (qRT‒PCR)

cDNA was synthesized from 300 ng of total RNA from microvessels using the High-Capacity cDNA Reverse Transcription Kit according to the manufacturer’s instructions. The synthesized cDNA samples were stored at -20 °C for later use. Real-time PCR was carried out by using a QuantStudio™ 3 Real-Time PCR System (Thermo Fisher Scientific; Waltham, USA).

qRT‒PCR was performed by using the Qiagen Mouse Endothelial Cell Biology RT2 Profiler PCR Array (PAMM-015Z) in combination with RT2 SYBR^®^ Green qPCR Mastermix (Qiagen, USA) according to the manufacturer’s recommendations [44, 45].

### Serum proinflammatory markers

Multiplex detection of serum proinflammatory markers was performed using the V-PLEX Proinflammatory Panel 1 Mouse Kit (MSD) (Cat No: K15048D-1; Meso Scale Discovery, Rockville, USA) according to the manufacturer’s instructions [46]. The assay allowed for the measurement of keratinocyte chemoattractant (KC)/human growth-regulated oncogene (GRO) (KC/GRO), TNF-α, interferon gamma (IFN-γ), IL-1β, interleukin-2 (IL-2), interleukin-4 (IL-4), interleukin-5 (IL-5), IL-6, interleukin-10 (IL-10), and interleukin-12 p70 (IL12p70). Data from V-PLEX Meso Scale experiments were analyzed based on standard curves included in the respective assay programs using MSD Discovery Workbench software (DISCOVERY WORKBENCH version 4.0.13).

### Mouse P-glycoprotein (P-gp) and P2 × 7R levels

Plasma P-glycoprotein (P-gp) levels were determined using a kit-based protocol according to the manufacturer’s instructions (MBS450526, MyBioSource, San Diego, USA). P2 × 7R was detected using a mouse purinergic P2 × 7R ELISA kit (Cat. No. E12339m-American Research Products, Waltham, MA, USA) with some modifications [16]. Circulating P2 × 7R levels were detected in serum samples collected at the end of harvest. The absorbance was measured at 450 nm using a microplate reader (SpectraMax^®^ M5).

### Plasma EV isolation and nanoparticle tracking analysis

EVs from plasma samples were isolated according to a kit-based protocol (cat. no. 4484450; Invitrogen, USA) [47]. Nanoparticle tracking analysis (NTA) of isolated EVs was performed using the NanoSight NS300 system fixed with a 488 nm laser (Malvern Technologies, Malvern, UK). Briefly, EV samples were diluted (1:500) in 1 mL of particle-free Milli-Q water (Milliporesigma, Burlington, USA) and injected into the NanoSight chamber using a 1 mL BD slip-tip syringe (Cat. No. 309659, Franklin Lakes, USA). Prior to running the samples, the machine was calibrated using 100 nm latex beads from Malvern, United Kingdom (Cat. No. NTA4088). The data were analyzed by NTA 3.3.104 software [16, 48].

### ATP detection in serum and EVs

Extracellular ATP levels in serum samples and EV suspensions were measured using the Luminescent ATP Detection Assay Kit from Abcam (Cat. No. ab113849, Cambridge, UK) in accordance with the manufacturer’s instructions with a few modifications. The EV suspension was subjected to sonication to lyse and then centrifuged at 10,000 rpm for 5 min [16]. Serum samples (35 µL) or EV supernatants (50 µL) were added to a Corning^®^ black clear bottom 96-well plate (Cat. No. 3603, Corning, USA) along with the standards. A total of 50 µL detergent was added to each well and incubated for 5 min at 600 rpm on an orbital shaker. Then, 50 µL of substrate was added to all the wells, followed by shaking at 600 rpm. The plates were covered and incubated in the dark for 10 min. Finally, luminescence was measured on an Infinite^®^ 200 M PRO (Tecan Austria GmbH).

### Western blot analysis

To characterize isolated EVs by western blot, we used EVs from six animals randomly of each cohort and checked for tetraspanin makers CD9 and CD81. The protein concertation of the isolated EVs was measured using the Thermo Scientific™ Pierce™ BCA Protein Assay Kit (Catalog No. PI23227, Thermo Fisher Scientific; Waltham, USA) and separated by polyacrylamide Mini-PROTEAN TGX gels (precast 4–20%) (Bio-Rad Laboratories, Hercules, CA, USA) according to the manufacturer’s protocol. Followed by electroblotting, nitrocellulose membranes were blocked with intercept blocking buffer (LI-COR, Lincoln, NE, USA) and incubated overnight with primary antibodies (dilution 1:1000), later probed with near-infrared secondary antibodies (LI-COR) (dilution 1:5000) and visualized with an Odyssey imaging system (LI-COR). The following primary anti-rabbit antibodies were used: CD9 (D8O1A) (1:1000, Cell Signaling Technology, Rabbit mAb #13174) and CD81 (D3N2D) (1:1000, Cell Signaling Technology, Rabbit mAb #56039) [16, 49].

### EV DNA isolation

To remove any DNA affixed to the EV surface, 100 µL of the EV suspension was treated with 10 U of DNase (LGC Biosearch Technologies, Cat. No. DB0715K, Hoddesdon, UK) for 20 min at 37 °C. DNase activity was stopped by the addition of 10 µL of 10X DNase stop solution. Following further dilution with 100 µL of nuclease-free water (NFW), the resultant EV suspension was lysed at room temperature using 20 µL of proteinase K (Cat. no. 4485229, Thermo Fisher Scientific; Waltham, USA). The DNeasy^®^ Blood & Tissue Kit from Qiagen (Cat. no. 69506, Hilden, DE) was used to isolate DNA from this EV suspension [16, 50, 51].

### EV mtDNA quantification by digital PCR

The isolated EV-DNA was diluted to a working concentration of 1 ng/µL with NFW. Mitochondrial gene-specific Taqman™ probes for ATP8 [mt-*Atp8*] (Cat. no. 4331182 Mm04225236_g1), NADH dehydrogenase 2 [mt-*Nd2*] (Cat. no. 4331182 Mm04225288_s1), cytochrome c oxidase subunit II [mt-*Cox2*] (Cat. no. 4331182 Mm03294838_g1), and 16 S ribosomal RNA [mt-*Rnr2*] (Cat. no. 4331182 Mm04260181_s1) were used in this experiment [51] (Thermo Fisher Scientific; Waltham, USA). PCRs were performed using 2 µL of 5X Absolute Q™ DNA Digital PCR Master Mix (Cat. no. A52490), 2 µL EV-DNA template (2 ng), 0.5 µL FAM-Taqman™ probe, and 5.5 µL NFW. A total of 9 µL of the above reaction mixture was loaded onto the QantStudioTMMAP16 Digital PCR plate (Cat. no. 10246917). Following the addition of 15 µL QuantStudioTM Absolute QTM Isolation Buffer (Cat. no. A52730) to each sample, the wells were sealed using gaskets that were provided with the dPCR plates. The PCR for mtDNA dPCR was as follows: 10 min at 96 °C, followed by 40 cycles of 5 s at 96 °C and 15 s at 60 °C. The QuantStudio™ Absolute Q Digital PCR System and QuantStudio dPCR software were used for DNA amplification, and the number of microchambers with successful mtDNA amplification was counted.

### Statistical analysis

Statistical analyses were performed utilizing Prism v10.3.1 software (GraphPad Software Inc., La Jolla, CA). *p* ≤ 0.05 was considered to indicate statistical significance. The results are expressed as the mean ± SEM. The significance between the groups in BECs analysis was assessed using Student’s t test. One-way analysis of variance (ANOVA) with Tukey’s post hoc test was performed for multiple group comparisons for serum cytokine levels, gene expression and EV numbers and ATP concentration in EVs [18]. Two-way ANOVA with Šídák’s multiple comparisons test analysis was performed for the P2 × 7R ELISA, P-gp and ATP release.

## Results

### BECs

Mice were exposed to ethanol vapors 4 days per week (16 h/day) to ensure that pathophysiologically relevant BECs were generated and maintained throughout the experiment. The observed BECs were 154.98 ± 10.70 mg/dL, 161 ± 10.19 mg/dL, 223.59 ± 20.15 mg/dL, and 259.73 ± 13.73 mg/dL in the CIE of the pharmacologically P2 × 7R-inhibited cohort, BBG-CIE, CIE of the P2 × 7R^−/−^ cohort, and CIE-P2 × 7R^−/−^ groups, respectively (Fig. 1B and C). We observed significantly higher BEC in the P2 × 7R^−/−^ mice cohort using the same ethanol exposure procedure.

### Effect of alcohol and P2 × 7R inhibition on gene expression in brain microvessels

**Fig. 2:**
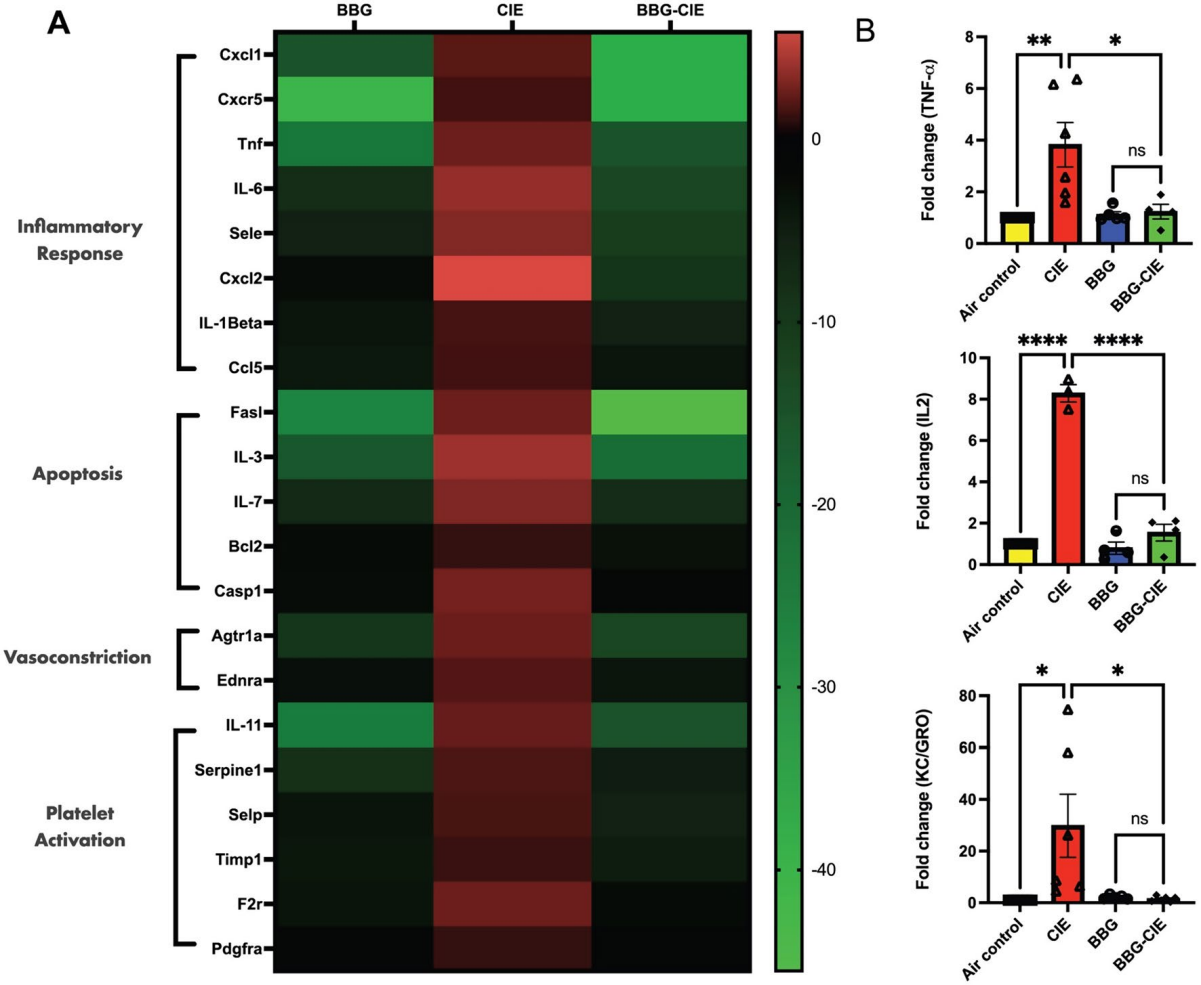
P2 × 7R inhibition reduced the expression level of genes involved in inflammation, apoptosis, vasodilation, and platelet activation in brain microvessels and serum cytokine levels in the BBG-treated–CIE-exposed animals. (**A**) Heatmap shows the upregulation of genes involved in inflammation, apoptosis, vasodilation, and platelet activation in the brain microvessels of the CIE-exposed animals. BBG treatment led to significant reduction in these levels. (**B**) Cytokine levels after CIE exposure were analyzed by MSD ELISA. The levels of the proinflammatory cytokines TNF-α, KC/GRO, and IL-2 were significantly lower in the BBG-treated–CIE-exposed animals than in the CIE-exposed animals only. One-way ANOVA followed by Tukey’s post hoc test was used for the statistical analyses; * *p* ≤ 0.05, **p ≤ 0.005, **** *p* < 0.0001 compared with CIE-exposed mice as controls. (*n* = 3–6, mean ± SEM)

Several studies have used brain microvessels to study the BBB and inflammation in vitro and ex vivo [52, 53]. CIE exposure significantly upregulated the expression profile of genes associated with inflammation (*Cxcl1*,* Il1b*,* Cxcr5*,* Tnf*,* Il6*,* Sele*,* Cxcl2*,* and Ccl2*), apoptosis (*Fasl*,* Iil3*,* Bcl2*,* Casp1*, and *Il7*), vasodilation (*Ednra and Agtr1a*), and platelet activation (*Serpine1*,* Selp*,* Timp1*,* Il11*,* F2r*, and *Pdgfra*) in the brain microvessels. P2 × 7R inhibition significantly reduced the CIE-induced neuroinflammatory response by 12–50-fold in the BBG-treated–CIE-exposed group (Fig. 2A; Table 1).

### Modulation of serum cytokine levels by P2 × 7R inhibition

Earlier studies have shown that alcohol-induced neuroinflammation results in increased expression of proinflammatory cytokines, such as TNFα, IL-1β, and IL6 [54]. Analysis of serum cytokine levels using MSD ELISA revealed a significant increase (2–30-fold) in proinflammatory cytokine levels in the CIE-exposed animals. A notable reduction in the serum levels of proinflammatory cytokines was observed after P2 × 7R suppression by BBG in the CIE-exposed animals. Significant decreases in TNF-α, KC/GRO, and IL-2 levels were detected in the BBG-treated–CIE-exposed animals compared with the CIE-exposed animals (Fig. 2B). Although not reaching statistical significance, the IL-1β, IFN-γ, and IL-5 levels also exhibited a decreasing trend in the BBG-treated animals. A greater level of IL-10 was detected in the BBG-treated–CIE-exposed animals than in the CIE-exposed animals (data not shown).

**Table 1:** Fold changes in the expression of genes involved in inflammation, apoptosis, vasodilation, and platelet activation in the brain microvessels of BBG, CIE- exposed, and BBG-treated–CIE-exposed mice. The observed values are normalized against the air control group

			Fold regulation		
Pathway	Gene	Gene	BBG	CIE-Exposed	BBG-CIE
Inflammatory response	*Cxcl1​*	Chemokine (C-X-C motif) ligand 1	-15.18	2.00​	-36.23​
	*Cxcr5​*	Chemokine (C-X-C motif) receptor 5	-40.22	1.45​	-35.94​
	*Tnf​*	Tumor necrosis factor	-22.57	2.49​	-15.18​
	*Il6​*	Interleukin 6	-7.99	3.59​	-12.50​
	*Sele​*	Selectin, endothelial cell	-5.45	3.16​	-11.05​
	*Cxcl2​*	Chemokine (C-X-C motif) ligand 2	-1.81	5.86​	-9.42​
	*Il1b​*	Interleukin 1 beta	-2.96	1.49​	-5.49​
	*Ccl5​*	Chemokine (C-C motif) ligand 5	-3.81	1.46​	-3.08​
Apoptosis	*Fasl​*	Fas ligand (TNF superfamily, member 6)	-26.13	2.48​	-45.54​
	*Il3​*	Interleukin 3	-15.8	3.90​	-20.45​
	*Il7​*	Interleukin 7	-7.12	2.97​	-7.64​
	*Bcl2​*	B-cell leukemia/lymphoma 2	-1.93	1.16​	-2.75​
	*Casp1​*	Caspase 1	-1.81	2.69​	-1.54​
Vasoconstriction	*Agtr1a​*	Angiotensin II receptor, type 1a	-9.76	2.46​	-12.56​
	*Ednra​*	Endothelin receptor type A	-2.65	1.88​	-2.96​
Platelet activation	*Il11​*	Interleukin 11	-23.9	2.33​	-15.05​
	*Serpine1​*	Serine (or cysteine) peptidase inhibitor, clade E, member 1	-8.53	1.67​	-4.54​
	*Selp​*	Selectin, platelet	-3.23	1.62​	-5.52​
	*Timp1​*	Tissue inhibitor of metalloproteinase 1	-3.59	1.28​	-4.75​
	*F2r​*	Coagulation factor II (thrombin) receptor	-3.21	2.49​	-1.85​
	*Pdgfra​*	Platelet derived growth factor receptor, alpha polypeptide	-1.24	1.16​	-1.09​
Other genes	*Nppb​*	Natriuretic peptide type B	-31.28	3.04​	-51.23​
	*Plg​*	Plasminogen	-46.47	1.25​	-48.29​
	*Mmp1a​*	Matrix metallopeptidase 1a (interstitial collagenase)	-21.65	6.25​	-25.97​
	*Tymp​*	Thymidine phosphorylase	-31.65	1.85​	-4.82​
	*Pgf​*	Placental growth factor	-7.00	1.67​	-2.86​
	*Mmp9​*	Matrix metallopeptidase 9	-2.75	1.58​	-1.59​
	*Tfpi​*	Tissue factor pathway inhibitor	-3.61	2.57​	-1.12​
	*Kit​*	Kit oncogene	1.21	1.46​	-1.66​
	*Angpt1​*	Angiopoietin 1	-3.04	1.45​	-1.39​
	*Plau​*	Plasminogen activator, urokinase	-3.30	1.51​	-1.08​

### P2 × 7R levels

**Fig. 3:**
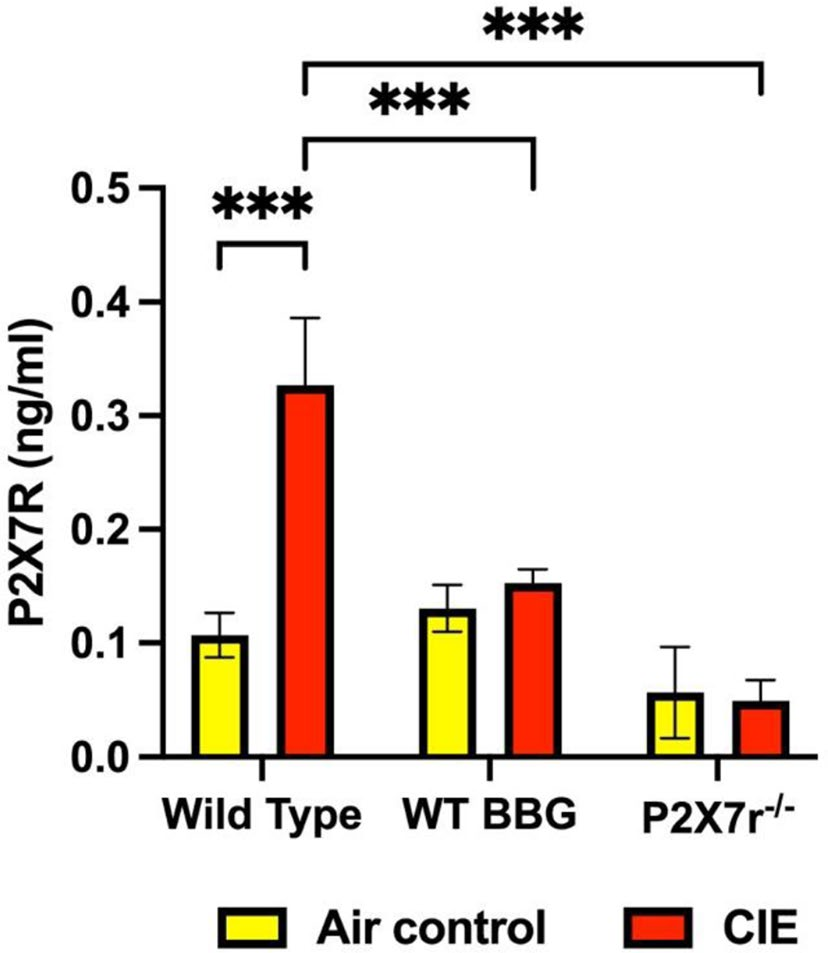
Serum levels of P2 × 7R upregulated by CIE were suppressed by receptor inhibition. P2 × 7R shedding was significantly less in the serum in BBG-treated–CIE-exposed or P2 × 7R^−/−^ CIE-exposed mice than in the serum of the CIE-exposed mice. Each bar represent mean ± SEM (*n* = 3–10), ***, *p* < 0.000. Two-way ANOVA was applied to P2 × 7R [interaction, F (2, 31) = 5.916, *p* = 0.0067; raw factor, F (2, 31) = 10.19, *p* = 0.0004; column factor, F (2, 31) = 10.19, *p* = 0.0224]

P2 × 7R shedding has been implicated in chronic inflammation and neurodegenerative diseases [32, 55, 56]. Earlier in the in vitro study, we found enhanced P2 × 7R shedding after EtOH exposure [17]. In vivo CIE exposure increased serum P2 × 7R levels by 2–4-fold compared with the air control group. BBG treatment or P2 × 7R knockout significantly reduced P2 × 7R shedding in the CIE-exposed mice **(**Fig. 3**)**.

### P-glycoprotein (P-gp)

**Fig. 4:**
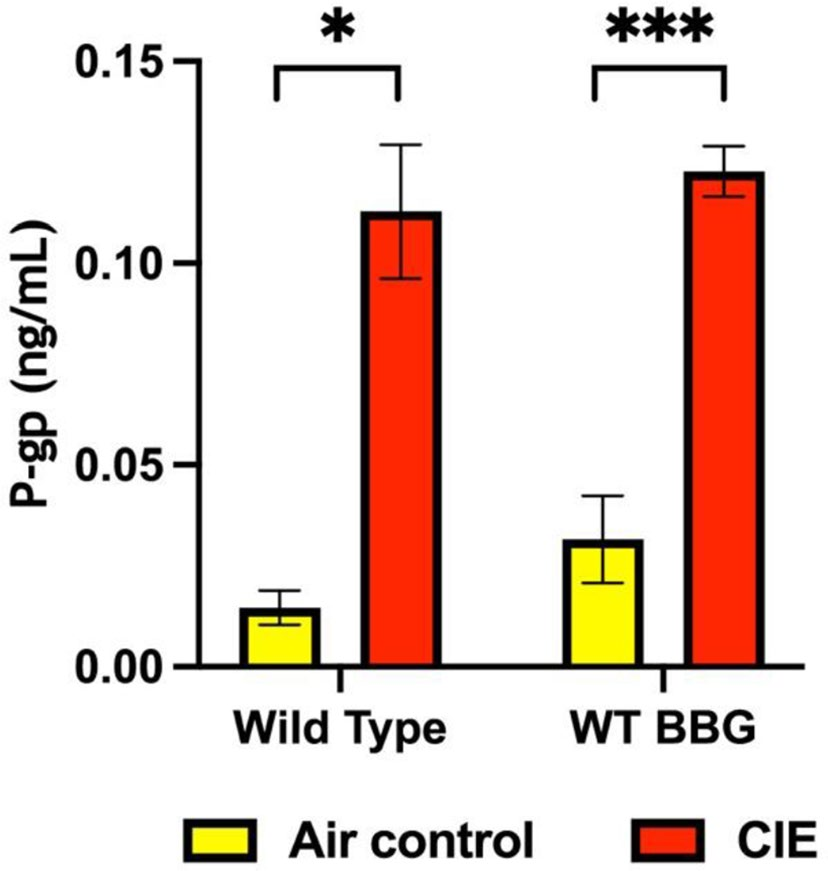
CIE exposure resulted in increased blood levels of P-glycoprotein (P-gp). The levels of plasma P-gp after CIE exposure were analyzed by ELISA. Plasma P-gp levels were significantly higher in the CIE-exposed mice than in air-control mice. Treatment with BBG had no effect on P-gp levels in the BBG-treated–CIE-exposed animals. Each bar represent mean ± SEM (*n* = 4–5), *p = 0.01, ***p < 0.0001. Two-way ANOVA analysis was applied to P-gp [interaction, F (1, 14) = 0.1159, *p* = 0.7386; raw factor, F (1, 14) = 1.1689, *p* = 0.2147; column factor, F (1, 14) = 83.91, *p* < 0.0001]

P-gp, an ATP-binding cassette subfamily B member 1 (ABCB1), plays a crucial role in BBB function and is involved in the efflux of toxic compounds back to the bloodstream [57]. It is only expressed on the brain endothelium; therefore, its increase in blood indicates BBB injury. Plasma P-gp levels were significantly higher in the CIE-exposed mice than in the air-control mice. BBG treatment did not alter P-gp levels in the CIE-exposed animals **(**Fig. 4**)**.

### Serum ATP and EV-ATP levels

**Fig. 5:**
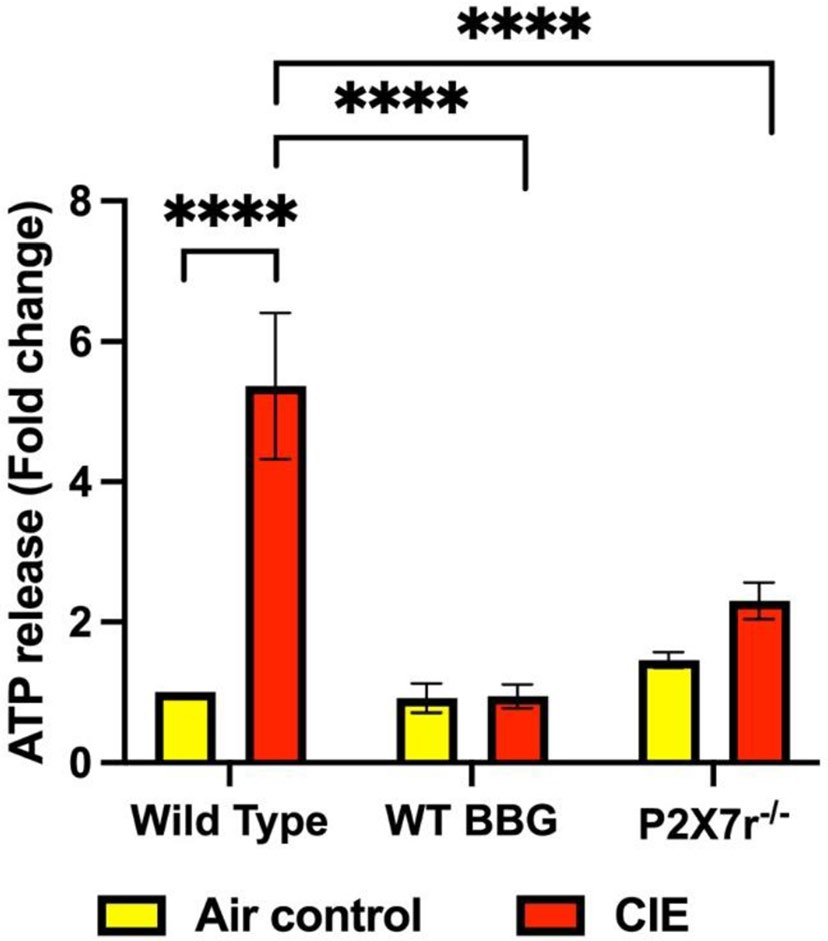
Serum ATP levels in the CIE exposed animals were normalized by pharmacologic or genetic P2 × 7R inhibition. Serum ATP levels were lower in the BBG-treated–CIE-exposed and P2 × 7R^−/−^ CIE-exposed mice than in the CIE-exposed mice. Statistics were carried out on fold change values (*n* = 5–7), **** *p* < 0.0005. Two-way ANOVA was applied to ATP release fold change values [interaction, F (2, 29) = 9.921, *p* = 0.0005; raw factor, F (2, 29) = 9.416, *p* = 0.007; column factor, F (1, 29) = 15.39, *p* = 0.0005]

P2 × 7R overactivation increases ATP concentrations [58]. CIE exposure increased serum ATP levels by 2-fold (from 0.26 µM to 0.48 µM) in the CIE exposed mice, whereas the BBG-treated–CIE-exposed mice showed serum ATP level reduced to 0.24 µM. In the case of CIE-P2 × 7R^−/−^ cohort, the serum ATP levels were 0.67 µM in the control CIE group, which was reduced to 0.21 µM in the P2 × 7R^−/−^ group (Fig. 5).

**Fig. 6:**
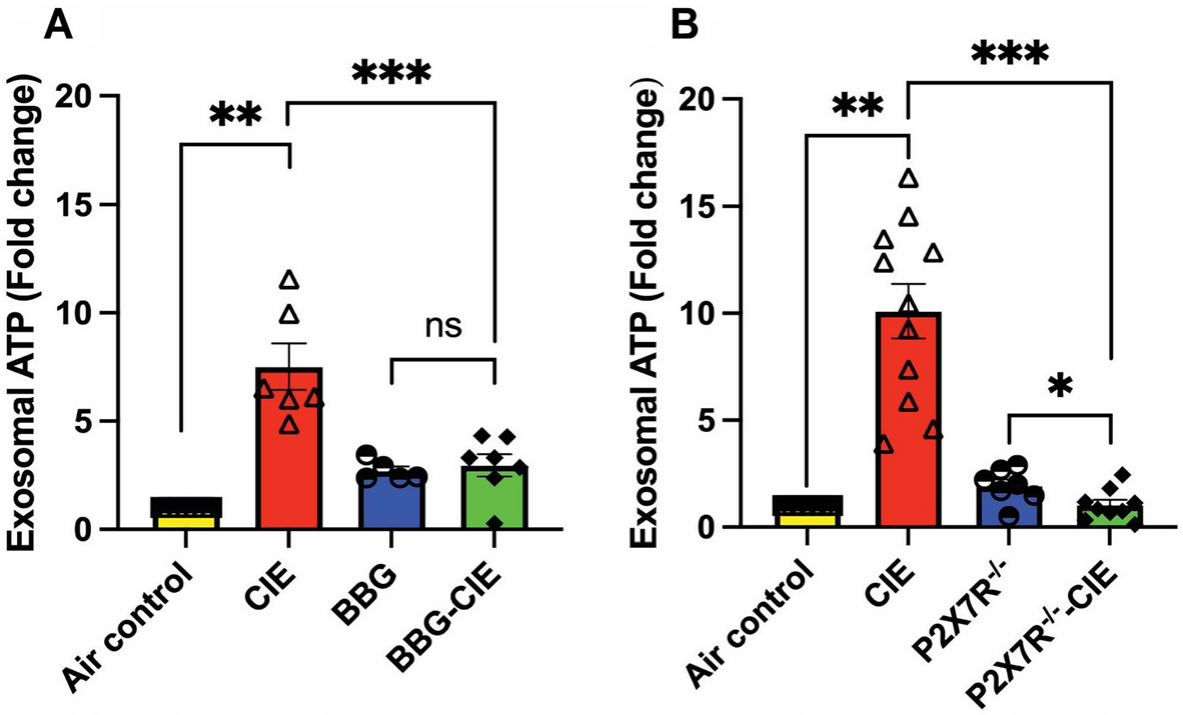
EV-ATP content increased in CIE animals were diminished by pharmacologic or genetic P2 × 7R inhibition. The serum EV-ATP levels, upregulated 7–10-fold by CIE, were reduced in (**A)** BBG-treated–CIE-exposed and (**B)** P2 × 7R^−/−^ CIE-exposed mice. One-way ANOVA followed by Tukey’s post hoc test was used for the statistical analyses. *n* = 5–7, mean ± SEM and *p ≤ 0.05, **p < 0.01 and ***p < 0.001, ns = nonsignificant

EVs are known to carry variety of cargos, including ATP, which play crucial roles in regulating inflammatory responses and immune cell activation [21, 47]. Serum EV-ATP levels were upregulated 7–10-fold in CIE-exposed animals as compared to air control group. However, serum EV-ATP levels were significantly reduced in the BBG-treated–CIE-exposed mice and CIE-exposed P2 × 7R^−/−^ mice when compared to in CIE-exposed mice (Fig. 6A and B). ATP levels normalized to the EV numbers are shown in Table 2.

**Table 2:** ATP concentrations normalized to the EV numbers

	Average ATP concentration (µM) /million EVs			Average ATP concertation (µM) /million EVs
Air control	0.01208061		Air control	0.001043
CIE	0.03007586		CIE	0.00541942
BBG	0.0278009		P2 × 7R^−/−^	0.003861117
BBG-CIE	0.03406355		P2 × 7R^−/−^ -CIE	0.00227653

#### EV numbers

**Fig. 7:**
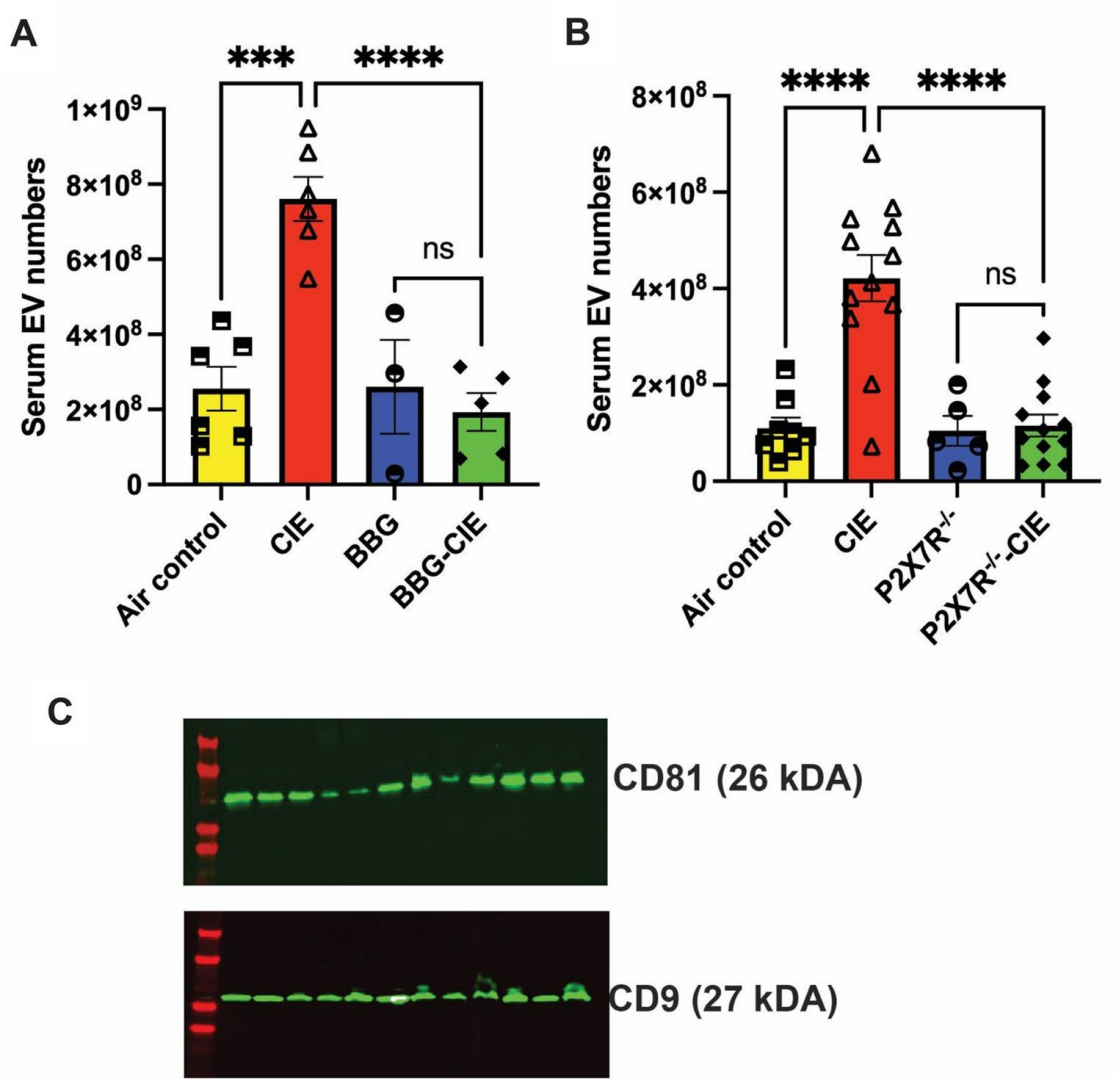
Genetic and pharmacologic P2 × 7R inhibition reduced EV numbers in the CIE-exposed mice. EV numbers were significantly lower in the **(A)** BBG-treated–CIE-exposed and **(B)** P2 × 7R^-/-^ CIE-exposed mice than in the respective CIE-exposed mice. **(C)** Validation of isolated EVs using tetraspanin markers CD9 and CD81 (*n* = 3). One-way ANOVA followed by Tukey’s post hoc test was used for the statistical analyses; ***p < 0.001, **** *p* < 0.0001, and ns = nonsignificant (*n* = 5–7, mean ± SEM)

EVs play critical roles in intercellular communication and can transport various bioactive molecules [59]. Studies have shown that chronic ethanol exposure results in increased number of enriched EVs in in vitro and in vivo [60, 61]. Here, we investigated the impact of P2 × 7R inhibition during CIE exposure on EV generation and found a 2–4-fold increase in the number of EVs (Fig. 7A and B). We found a significant reduction (*p* < 0.001) in the number of EVs in the BBG-treated–CIE-exposed and P2 × 7R^−/−^ CIE-exposed mice compared to their respective CIE-exposed controls. The isolated EVs were validated by the presence of tetraspanin markers, CD9 and CD81, using western blot (Fig. 7C).

### mtDNA copy numbers in EVs

**Fig. 8:**
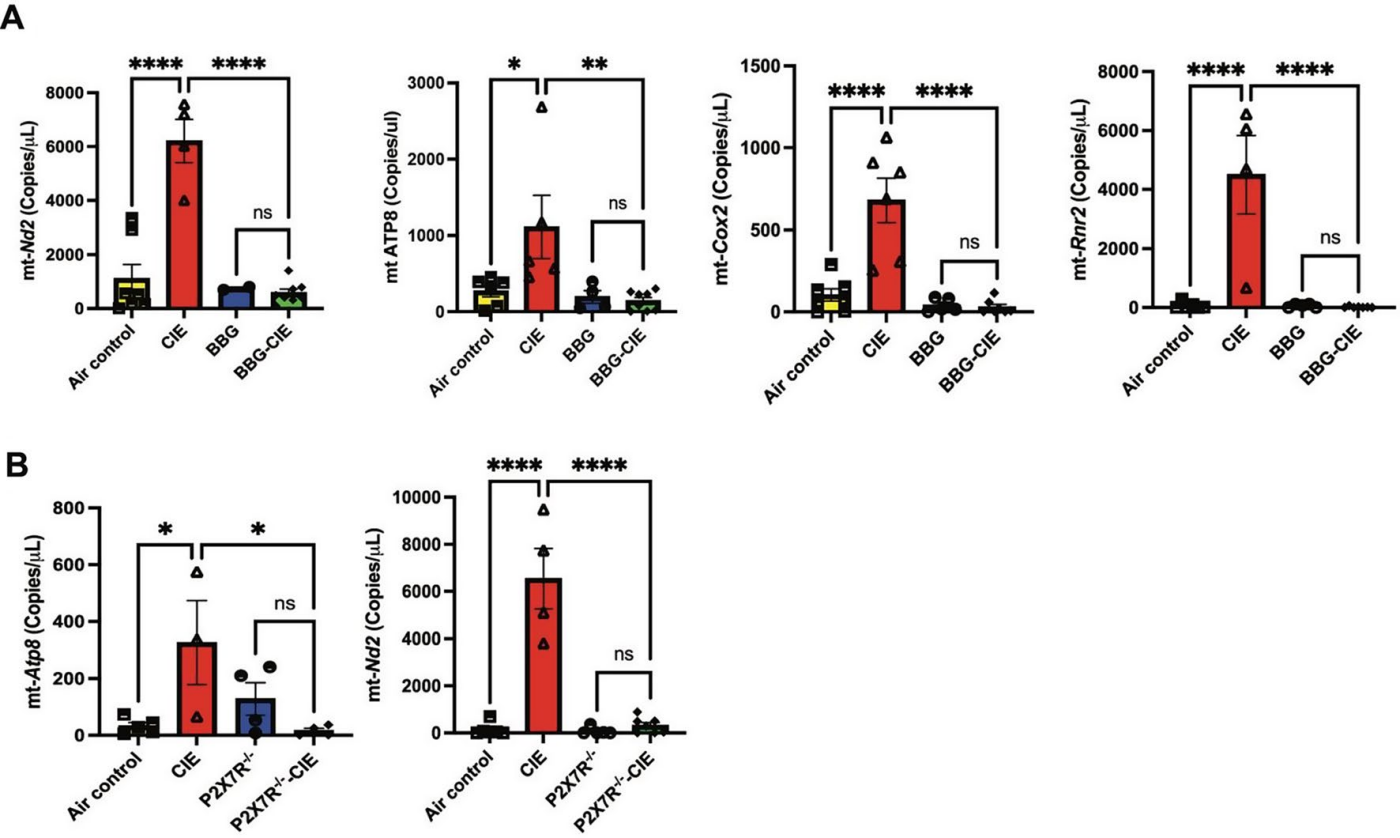
Genetic and pharmacologic P2 × 7R inhibition reduces mitochondrial gene expression, enhanced by CIE. Digital PCR was used to quantify the copy numbers of mtDNA present in EVs. Bar graphs represent the copy number of genes per microliter of DNA in the experimental groups. **(A)** P2 × 7R inhibition or **(B)** P2 × 7R^-/-^ reduces mtDNA copy numbers in the EVs isolated from BBG-treated–CIE exposed and P2 × 7R^-/-^ CIE-exposed animals compared to that of EVs from CIE-exposed animals. One-way ANOVA followed by Tukey’s post hoc test were used for the statistical analyses. mean ± SEM, * *p* ≤ 0.01, **p ≤ 0.001, **** *p* < 0.0001, and ns = nonsignificant. (*n* = 3–8)

Studies have shown that EVs isolated from ethanol-exposed cells have more damaged mtDNA, which acts as DAMPs and leads to the activation of autocrine and paracrine signaling pathways [62]. We utilized digital PCR to quantify the copy numbers of mtDNA present in EVs. We evaluated three genes (*mt-Nd2*,* mt-Atp8*,* mt-Cox2*) having crucial role in mitochondrial respiration and another gene *mt-RNR2*, which is a part of the mitochondrial ribosome. Compared to the air-control group, the CIE-exposed mice showed significantly higher EV mtDNA level, which was significantly reduced in the BBG-treated–CIE-exposed mice. Copy numbers of *mt-Nd2* and *mt-Atp8* were significantly lower in EVs from the P2 × 7R^-/-^CIE-exposed mice than in the EVs isolated from the CIE-exposed mice (Fig. 8A and B).

## Discussion

The present study sought to understand the role of P2 × 7R signaling in neuroinflammatory responses and BBB damage caused by CIE exposure in mice. We report, for the first time, that pharmacological blocking of P2 × 7R and genetic knockout of P2 × 7r may diminish CIE-induced BBB injury in vivo.

High BECs in CIE-exposed mice are indicative of pathophysiological relevant ethanol levels [35] in all mice **(**Fig. 1B **and C)**. All mice in the CIE-exposed group were exposed to ethanol vapors for 16 h per day. However, despite being exposed with the similar protocol, the observed BECs in the P2 × 7R knockout mice were significantly higher compared to wild-type mice **(**Fig. 1A**).** We speculate these differences might result from difference in the ethanol metabolism between the two groups.

Chronic alcohol consumption alters the peripheral immune profile, signaling peripheral organs and brain microglia and astrocytes to release pro-inflammatory cytokines [63]. Study by Lowe et al. has shown the increased expression of TNFα, IL-1β, and CCL2 along with other proinflammatory cytokines as the characteristic feature of alcohol-induced neuroinflammation [54]. Upon CIE exposure, we noted significant increase in the serum levels of TNF-α, KC/GRO, IL-2, IL-1β, IFN-γ, and IL-5. Notably, increased levels of these cytokines in the periphery are associated with alcohol use disorder in humans [64]. IFN-γ acts as potent inducer of TNF-α during neuroinflammation [65, 66]. The increased concentration of KC/GRO has been reported at the time of BBB damage [67]. Alcohol intoxication induced plasma IL-1β and IL-2 in rhesus macaques [68]. In the present study, levels of these cytokines were alleviated by pharmacological blockage and genetic knockout of P2 × 7R, suggesting its crucial role in the CIE-induced neuroinflammation (Fig. 2B). Similarly, Asatryan and collogues reported overactivation of the P2 × 7R and increased mRNA expression of proinflammatory cytokines IL-1β, TNF-α, and IL-6 in the mouse model of chronic EtOH exposure combined with high-fat diet [15, 69].

Studies have shown that BBB dysfunction amplifies neuroinflammation [8]. Brain microvessels serve as an excellent ex vivo model to study the BBB [52, 53, 70, 71]. Targeted blockade of P2 × 7R serves as a potential path to combat neuroinflammation [72–76]. In experimental autoimmune encephalomyelitis, BBG-dependent P2 × 7R antagonism resulted in decreased BBB damage with normalized levels of PDGFβR and claudin-5 and pro-inflammatory cytokines, IL-1β, IL-6, and TNF-α [77, 78]. Earlier studies have shown the crucial role of the CCR2/5 axis in peripheral macrophage recruitment into the CNS and in microglial alterations upon chronic ethanol consumption in mouse models [54]. Elevated CCL2 production via MCP-1/CCR2-mediated pathway was followed by P2 × 7R activation in the brain [79]. P2 × 7R is involved in the caspase-1 activation, leading to increased IL-1β and TNF-α levels, which causes apoptosis [80]. We found that BBG-induced P2 × 7R blockade resulted in a 2–50-fold downregulation of genes associated with inflammation (*Cxcl1*,* Il1b*,* Cxcr5*,* Tnf*,* Il6*,* Sele*,* Cxcl2*,* and Ccl2*), apoptosis (*Fasl*,* Iil3*,* Bcl2*,* Casp1*, and *Il7*), vasodilation (*ednra and agtr1a*), and platelet activation (*Serpine1*,* Selp*,* Timp1*,* Il11*,* F2r*, and *Pdgfra*) (Table 1; Fig. 2A). These changes in gene expression in the BBG-treated–CIE-exposed mice underscore the significance of P2 × 7R inhibition on the transcriptional landscape within brain microvessels during chronic EtOH exposure.

In chronic inflammation and neurodegenerative diseases, enhanced P2 × 7R shedding has been observed [32, 55, 56]. Additionally, in vitro dendritic cell P2 × 7R stimulation with ATP can trigger shedding of microvesicles carrying the P2 × 7R itself, suggesting a regulatory role of P2 × 7R signaling in its own shedding process [81]. Earlier, we showed that in vitro treatment of lung epithelial cells with EtOH increased P2 × 7R shedding [17]. In this study, we observed a substantial increase in the circulatory P2 × 7R levels in the CIE-exposed animals, which was significantly reduced by pharmacologic or genetic P2 × 7R inhibition (Fig. 3). The observed results add to the growing body of evidence, implicating P2 × 7R shedding in the regulation of P2 × 7R signaling and activity.

P-gp is an efflux transporter with a crucial role in the transport of substances across the BBB. Chronic alcohol exposure significantly increases P-gp mRNA and protein expression in vitro [82]. TNF-α and endothelin-1 exposure also stimulates P-gp expression [83]. We found a significant increase in P-gp levels in the blood of CIE-exposed mice, reflecting BBB injury. However, treatment with BBG did not alter P-gp levels in the CIE-exposed animals, suggesting that P2 × 7R signaling may not regulate P-gp expression (Fig. 4). More recently, Arnaud-Sampaio and colleagues have shown that P2 × 7R isoform B has higher efflux activity than P2 × 7R A, which may be mediated by P-gp and other ABC transporters [84].

P2 × 7R are ATP-gated cation channel receptors and undisputedly serve as gatekeepers of inflammation [73, 85]. P2 × 7R-dependent ATP release contributes to increased eATP levels in osteoclast and osteoblast cultures, highlighting autocrine/paracrine role of P2 × 7R signaling [86]. Studies have highlighted the importance of P2 × 7R-mediated ATP release in initiating and amplifying inflammatory responses in the CNS and peripheral tissues [87]. Similarly, we found increased serum ATP level in the CIE-exposed mice. The BBG-treated–CIE-exposed or P2 × 7R^-/-^CIE-exposed mice exhibited significantly lower serum ATP levels compared to CIE-exposed mice (Fig. 5). This suggests that inhibition or genetic knockout of the P2 × 7R reduces eATP release, a neuroinflammatory messenger, mitigating neuroinflammatory responses associated with chronic alcohol exposure [88].

Several studies have reported that P2 × 7R stimulation by ATP triggers EV shedding with significant change in their size [19, 20]. Moreover, EV cargo composition in various cell types, including immune cells and neurons is influenced by P2 × 7R stimulation by ATP [21, 22, 89]. In the context of chronic alcohol exposure, limited research has explored the role of P2 × 7R in EV regulation and its implications in alcohol-induced pathology. Pfeiffer and colleagues have noted that ATP-dependent P2 × 7R activation results in P38-MAPK-facilitated cytoskeletal restructuring, leading to EV release [90]. We found a drastically increased number of EVs in the CIE-exposed mice, which was significantly lowered in both BBG-treated and P2 × 7R^-/-^ CIE-exposed mice (Fig. 7). The observed ATP values, when normalized to the EV numbers, showed elevated ATP levels in the CIE-exposed EVs. BBG treatment did not influence the ATP levels of EVs, but it did decrease EV numbers (Table 2). These data indicate a potential role of P2 × 7R in EV biogenesis and secretion due to alcohol exposure in vivo. The mechanism of increasing EV generation and their content changes are of considerable interest to investigate.

Interestingly, Ibáñez and colleagues have reported EtOH-induced EV secretion with significant alterations in lipid metabolism and EV enrichment with inflammatory-related proteins and miRNAs in BV2 microglia and astrocytes [23, 91]. Studies have reported changes in EV composition after alcohol exposure in liver and lung cells [92, 93]. EV-mediated ATP signaling plays an important role in regulating inflammatory responses and immune cell activation [94]. Along with other components, ATP itself is present in released EVs [21, 95]. Our study showed reduced serum EV-ATP levels following P2 × 7R inhibition or knockout in the CIE-exposed mice (Fig. 6), potentially mitigating the pro-inflammatory and pathological effects associated with chronic alcohol exposure.

EVs can cross BBB, carry exchange between the CNS and blood, and regulate neuroinflammation [96–98]. P2 × 7R overactivation leads to mitochondrial damage, causing the release of mitochondrial content, including Ca^2+^, ATP, and mtDNA into the extracellular environment [16, 99]. Chronic alcohol exposure of hepatocyte causes release of EVs, containing mtDNA fragments, which act as DAMPs with proinflammatory properties, activating autocrine and paracrine signaling pathways [100]. Of note, EtOH-induced mtDNA damage is involved in the release of exosomes enriched with damaged mtDNA in vitro [62]. Recent reports have demonstrated the presence of mtDNA fragments in hepatocyte-derived EVs after EtOH-exposure in vivo and in patients with fatty liver conditions [24, 25]. Our prior study showed that P2 × 7R inhibition reduces mtDNA copy numbers in EVs from EtOH-treated lung epithelial cells in vitro [17]. In this study, we report a significant increase in mtDNA copy numbers in isolated EVs from the CIE-exposed mice (Fig. 8A, B), whereas P2 × 7R inhibition or knockout significantly lowered mtDNA content in the CIE-exposed mice. It is known that cytosolic mtDNA-mediated NLRP3 inflammasome activation is associated with caspase-1 activation and IL-1β/IL-18 production [101, 102]. The increased expression of caspase-1 and IL-1β (Fig. 2A; Table 1) in isolated brain microvessels further supports this observation. Additionally, mtDNA activates TLR9-MyD88 downstream signaling, causing NF-κB activation, which triggers the production of pro-inflammatory cytokines and chemokines [103, 104]. Increased mtDNA concentration in isolated EVs and significantly increased gene expression of *Tnf-α*,* Il-6*,* Il-1β*,* Cxcl1*,* Cxcl2*, and *Cxcr5* from EtOH-exposed groups corroborate these findings (Fig. 2A; Table 1). In earlier studies, the presence of mtDNA in EVs was detected using gene-specific probes for mt-*Nd2*, mt-*Atp8*, mt-*Cox2*, and mt-*Rnr2* [50, 105]. These genes are involved in the mitochondrial respiration [106]. In the present study, significantly increased mtDNA concentration in the EVs and proinflammatory changes in the periphery and BBB of the CIE-exposed group suggest that the mtDNA carried by EVs acts as DAMP and may cause an exacerbation of neuroinflammation [105, 107].

**Fig. 9:**
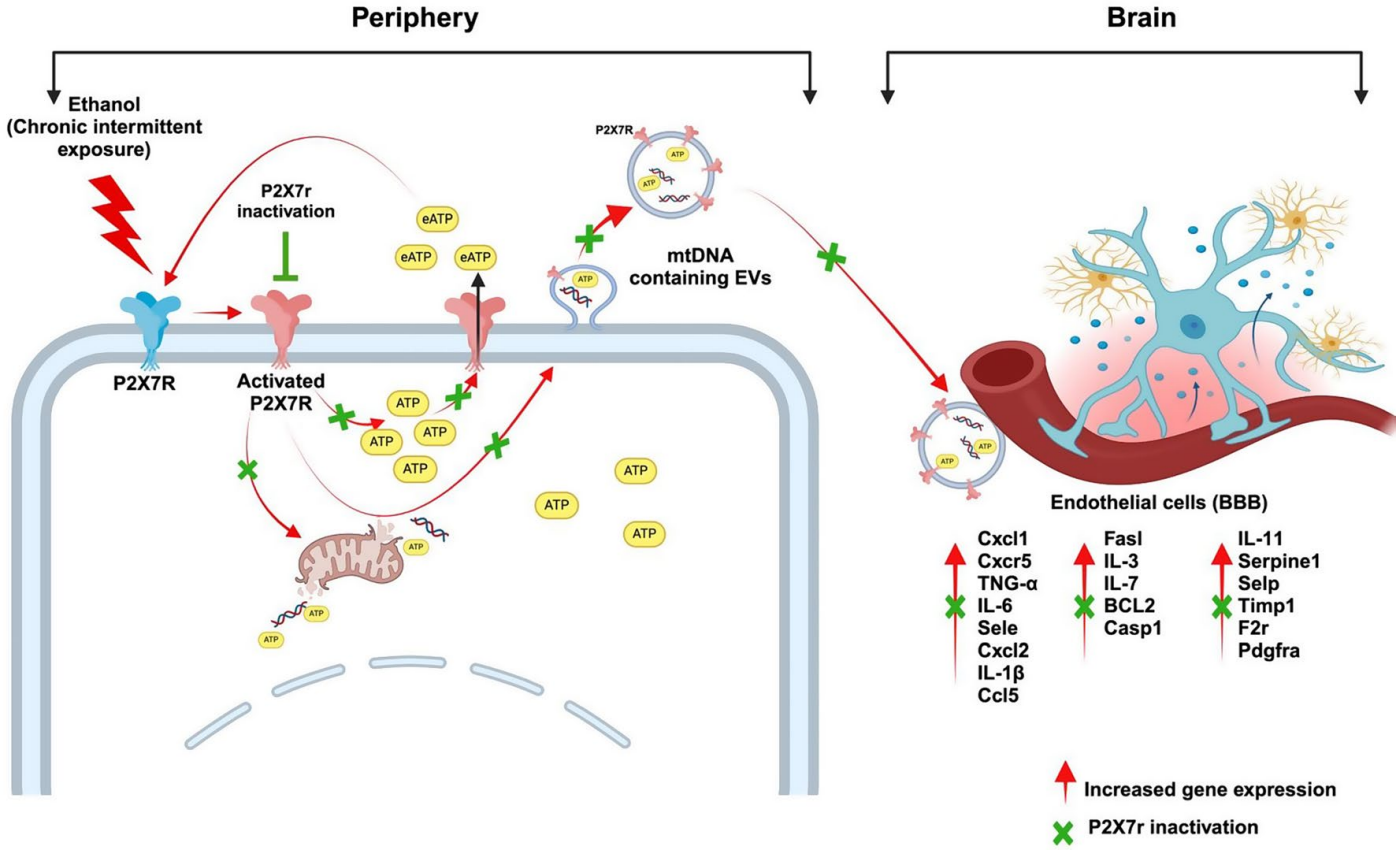
Schematic of CIE-induced P2 × 7R signaling promoting neuroinflammation. We summarize from this study that CIE leads to P2 × 7R activation, resulting in increased levels of extracellular ATP, EV-ATP, and a higher number of EVs. The released eATP further activates P2 × 7R through paracrine signaling, amplifying the response. The EVs also contain elevated mtDNA copy numbers, which may serve as DAMPs, contributing to inflammation in brain endothelial cells. P2 × 7R inactivation significantly reduces CIE induced responses, suggesting a novel mechanism of brain injury during CIE exposure via P2 × 7R signaling”. Figure created using Biorender.com

Our observations imply that P2 × 7R signaling may play a role in regulating EV release and their cargo contents. While the specific mechanisms underlying P2 × 7R-mediated regulation of mitochondrial stress and escape of mtDNA in EVs remain to be fully elucidated, our findings suggest a potential link between P2 × 7R signaling, mitochondrial dysfunction, and EV dynamics in the context of chronic alcohol exposure and neuroinflammation (Fig. 9). Future studies of the mechanism of EV release, link to P2 × 7R activation, and cross-organ communication by EVs may pave the way to future treatment interventions.

Although our study offers insightful information in several areas, we believe that there are few limitations that should be noted. To test for BBB pathology, we checked markers, including tight junction protein, claudin, and occludin, but we could not identify any discernible alterations in these markers [69, 108]. These discrepancies might have resulted from a variety of reasons, including subcellular localization of tight junction protein (without significant changes in their content), the exact scheduling and evaluation settings we selected, which were not ideal for picking up on minute changes. Further research employing a wider variety of markers and techniques will contribute to the clarification of potential alterations in brain tissue. Notwithstanding these drawbacks, our work adds to our knowledge of the critical role P2 × 7R signaling plays in CIE-induced neuroinflammation and emphasizes the significance of additional research to address these challenges and gain a broader understanding of the underlying P2 × 7R-mediated neuroimmune signaling.

## Conclusion

The present study delved into understanding the impact of P2 × 7R signaling on neuroinflammatory responses and BBB injury induced by CIE exposure. Blockade of P2 × 7R channels resulted in reduced eATP release, downregulation of genes associated with inflammation, apoptosis, vasodilation, and platelet activation, underscoring the critical role of P2 × 7R in CIE-induced neuroinflammation. Furthermore, inhibition or genetic knockout of P2 × 7R led to altered EV dynamics, such as reduced quantity and eATP and mtDNA levels, suggesting a potential regulatory role of P2 × 7R signaling in mitigating chronic alcohol-induced pro-inflammatory effects associated with EVs. These findings contribute to understanding the complex interplay between P2 × 7R signaling, peripheral and neuro-inflammation, BBB integrity, and circulating EV biology in the context of in vivo chronic alcohol exposure.

## Electronic supplementary material

Below is the link to the electronic supplementary material.

Supplementary Material 1

Supplementary Material 2

Supplementary Material 3

Supplementary Material 4

Supplementary Material 5

Supplementary Material 6

Supplementary Material 7

Supplementary Material 8

Supplementary Material 9

Supplementary Material 10

Supplementary Material 11

Supplementary Material 12

## Data Availability

No datasets were generated or analysed during the current study.

## Abbreviations

BBB: Blood‒brain barrier
EV: Extracellular vesicle
CIE: Chronic intermittent ethanol (CIE)
BBG: Brilliant Blue G
BEC: Blood ethanol concentration
eATP: Extracellular ATP
P7X7R: Purinergic receptor P2 × 7
DAMPs: Damage-associated molecular patterns
NLRP3: Nod-like receptor pyrin domain containing 3
EtOH: Ethanol
BMVECs: Brain microvascular endothelial cells
mtDNA: Mitochondrial DNA
mt-ATP-8: Mitochondrially encoded ATP synthase membrane subunit 8
mt-ND2: NADH dehydrogenase 2
mt-COX2: Cytochrome c oxidase subunit II
mt-RNR2: 16 S ribosomal RNA
HBSS: Hank’s balanced salt solution
P-gp: P-glycoprotein
KC/GRO: Keratinocyte chemoattractant (KC)/human growth-regulated oncogene (GRO)
TNF-α: Tumor necrosis factor alpha
IFN-γ: Interferon gamma
IL-1β: Interleukin 1 beta
IL-2: Interleukin-2
IL-4: Interleukin-4
IL-5: Interleukin-5
IL-6: Interleukin-6
IL-10: Interleukin-10
IL12p70: Interleukin-12 p70
