# Supplementary material for: Neuroinflammatory responses and blood–brain barrier injury in chronic alcohol exposure: role of purinergic P2 × 7 Receptor signaling
Source: J Neuroinflammation. 2024 Sep 28;21:244. doi: 10.1186/s12974-024-03230-4 (PMC11439317; doi:10.1186/s12974-024-03230-4)
Supplement: Supplementary file 1 — Supplementary Material 1 [file 12974_2024_3230_MOESM1_ESM.pdf]

NANOSIGHT

49 2023-12-06 13-59-39

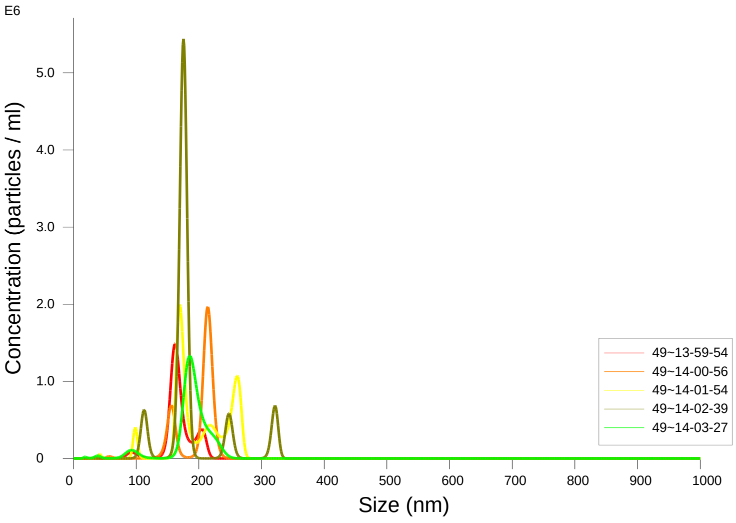

FTLA Concentration / Size graph for Experiment:
49 2023-12-06 13-59-39

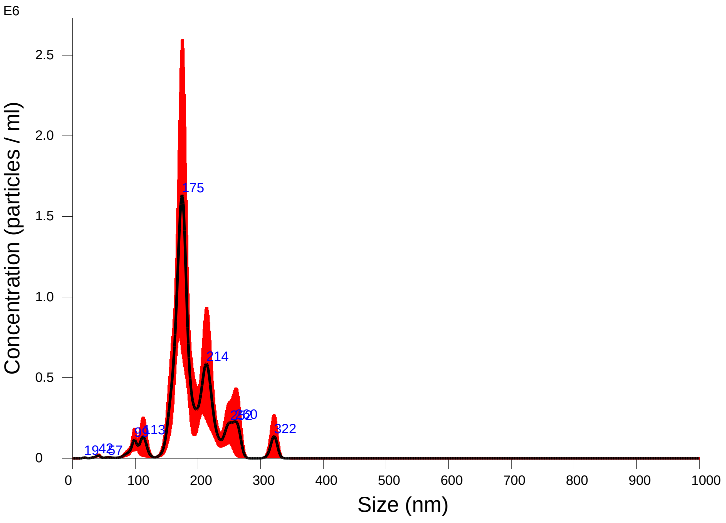

Averaged FTLA Concentration / Size for Experiment:
49 2023-12-06 13-59-39
Error bars indicate + / -1 standard error of the mean

Included Files

49 2023-12-06 13-59-54
49 2023-12-06 14-00-56
49 2023-12-06 14-01-54
49 2023-12-06 14-02-39
49 2023-12-06 14-03-27

Details

NTA Version:	NTA 3.3 Dev Build 3.3.104
Script Used:	SOP Standard Measurement 01-59-39PM 06~
Time Captured:	13:59:39 06/12/2023
Operator:	
Pre-treatment:	
Sample Name:	49
Diluent:	water
Remarks:	1:100

Capture Settings

Camera Type:	sCMOS
Laser Type:	Blue488
Camera Level:	11
Slider Shutter:	890
Slider Gain:	146
FPS	25.0
Number of Frames:	749
Temperature:	23.9 - 24.0 ºC
Viscosity:	(Water) 0.909 - 0.912 cP
Dilution factor:	Dilution not recorded

Analysis Settings

Detect Threshold:	7
Blur Size:	Auto
Max Jump Distance:	Auto: 11.1 - 26.4 pix

Results

Stats: Merged Data

Mean:	190.4 nm
Mode:	174.6 nm
SD:	42.1 nm
D10:	156.7 nm
D50:	179.9 nm
D90:	248.3 nm

Stats: Mean +/- Standard Error

Mean:	189.0 +/- 6.0 nm
Mode:	181.4 +/- 9.1 nm
SD:	36.3 +/- 4.7 nm
D10:	160.1 +/- 3.3 nm
D50:	186.8 +/- 7.7 nm
D90:	232.2 +/- 11.1 nm
Concentration (Upgrade):	6.44e+07 +/- 1.20e+07 particles/ml
	6.7 +/- 1.2 particles/frame
	8.8 +/- 1.3 centres/frame

Concentration measurements may be unreliable
See summary file for more info

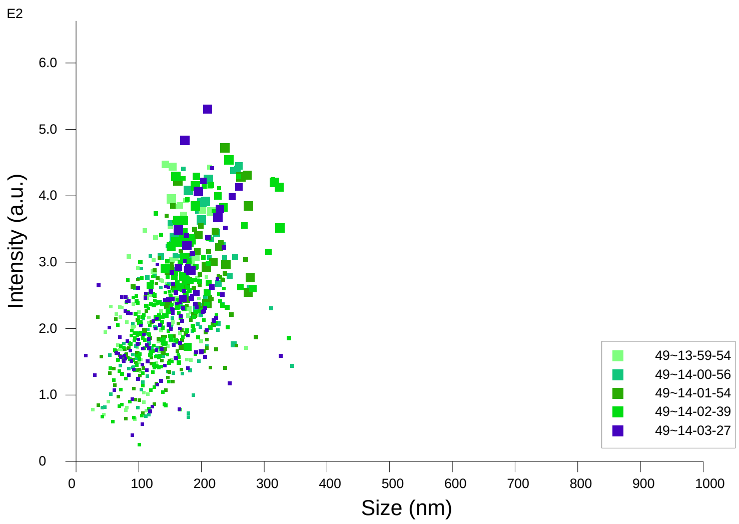

Intensity / Size graph for Experiment:
49 2023-12-06 13-59-39

Script Used: (Full Text):

SOP Standard Measurement 01-59-39PM 06Dec2023.txt
