# Supplementary material for: Neuroinflammatory responses and blood–brain barrier injury in chronic alcohol exposure: role of purinergic P2 × 7 Receptor signaling
Source: J Neuroinflammation. 2024 Sep 28;21:244. doi: 10.1186/s12974-024-03230-4 (PMC11439317; doi:10.1186/s12974-024-03230-4)
Supplement: Supplementary file 2 — Supplementary Material 2 [file 12974_2024_3230_MOESM2_ESM.pdf]

NANOSIGHT

45 2023-12-07 15-47-33

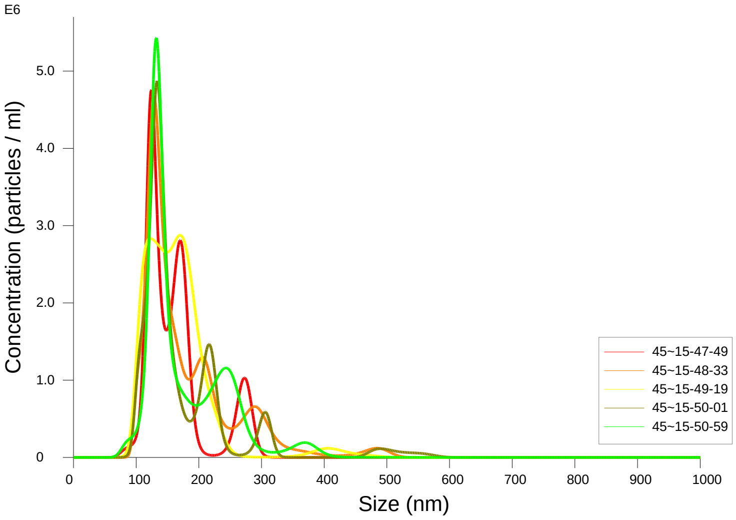

FTLA Concentration / Size graph for Experiment:
45 2023-12-07 15-47-33

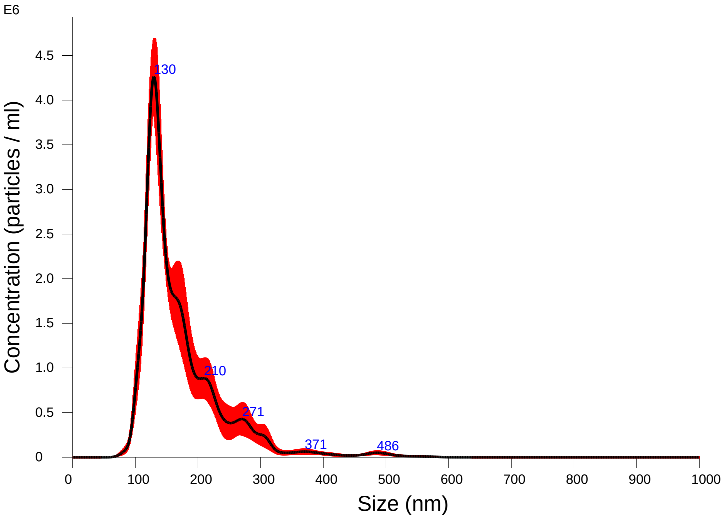

Averaged FTLA Concentration / Size for Experiment:
45 2023-12-07 15-47-33
Error bars indicate + / -1 standard error of the mean

Included Files

45 2023-12-07 15-47-49
45 2023-12-07 15-48-33
45 2023-12-07 15-49-19
45 2023-12-07 15-50-01
45 2023-12-07 15-50-59

Details

NTA Version:	NTA 3.3 Dev Build 3.3.104
Script Used:	SOP Standard Measurement 03-47-33PM 07~
Time Captured:	15:47:33 07/12/2023
Operator:	
Pre-treatment:	
Sample Name:	45
Diluent:	water
Remarks:	1:100

Capture Settings

Camera Type:	sCMOS
Laser Type:	Blue488
Camera Level:	10
Slider Shutter:	696
Slider Gain:	73
FPS	25.0
Number of Frames:	749
Temperature:	24.8 - 24.8 ^{o}C
Viscosity:	(Water) 0.892 - 0.893 cP
Dilution factor:	Dilution not recorded

Analysis Settings

Detect Threshold:	5
Blur Size:	Auto
Max Jump Distance:	Auto: 12.6 - 13.7 pix

Results

Stats: Merged Data

Mean:	171.0 nm
Mode:	129.7 nm
SD:	67.0 nm
D10:	115.9 nm
D50:	148.9 nm
D90:	257.5 nm

Stats: Mean +/- Standard Error

Mean:	170.7 +/- 3.6 nm
Mode:	137.7 +/- 8.3 nm
SD:	65.3 +/- 5.9 nm
D10:	115.9 +/- 1.5 nm
D50:	149.0 +/- 2.3 nm
D90:	252.7 +/- 13.0 nm
Concentration (Upgrade):	2.84e+08 +/- 1.19e+07 particles/ml
	21.7 +/- 1.4 particles/frame
	22.8 +/- 1.4 centres/frame

45 2023-12-07 15-47-33

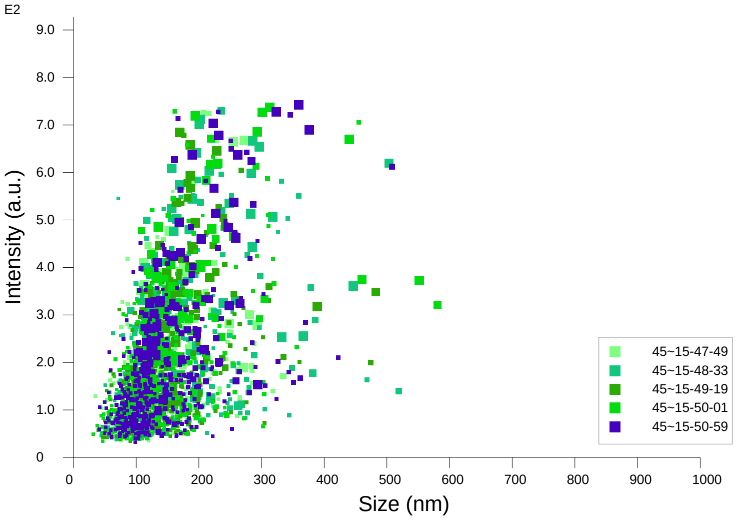

Intensity / Size graph for Experiment:
45 2023-12-07 15-47-33

Script Used: (Full Text):

SOP Standard Measurement 03-47-33PM 07Dec2023.txt
