# Supplementary material for: Neuroinflammatory responses and blood–brain barrier injury in chronic alcohol exposure: role of purinergic P2 × 7 Receptor signaling
Source: J Neuroinflammation. 2024 Sep 28;21:244. doi: 10.1186/s12974-024-03230-4 (PMC11439317; doi:10.1186/s12974-024-03230-4)
Supplement: Supplementary file 3 — Supplementary Material 3 [file 12974_2024_3230_MOESM3_ESM.pdf]

NANOSIGHT

81 2023-12-07 15-40-07

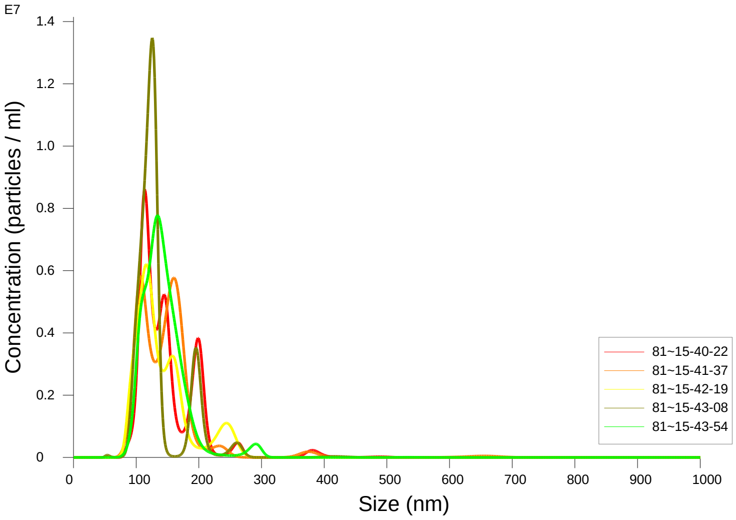

FTLA Concentration / Size graph for Experiment:
81 2023-12-07 15-40-07

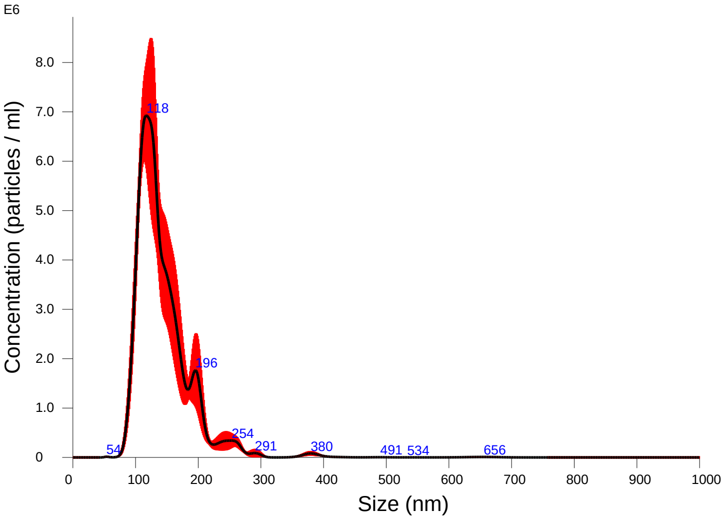

Averaged FTLA Concentration / Size for Experiment:
81 2023-12-07 15-40-07
Error bars indicate + / -1 standard error of the mean

Included Files

81 2023-12-07 15-40-22
81 2023-12-07 15-41-37
81 2023-12-07 15-42-19
81 2023-12-07 15-43-08
81 2023-12-07 15-43-54

Details

NTA Version:	NTA 3.3 Dev Build 3.3.104
Script Used:	SOP Standard Measurement 03-40-07PM 07~
Time Captured:	15:40:07 07/12/2023
Operator:	
Pre-treatment:	
Sample Name:	81
Diluent:	water
Remarks:	1:100

Capture Settings

Camera Type:	sCMOS
Laser Type:	Blue488
Camera Level:	10
Slider Shutter:	696
Slider Gain:	73
FPS	25.0
Number of Frames:	749
Temperature:	24.8 - 24.8 ^{o}C
Viscosity:	(Water) 0.892 - 0.893 cP
Dilution factor:	Dilution not recorded

Analysis Settings

Detect Threshold:	5
Blur Size:	Auto
Max Jump Distance:	Auto: 13.0 - 14.1 pix

Results

Stats: Merged Data

Mean:	143.3 nm
Mode:	117.2 nm
SD:	47.1 nm
D10:	103.1 nm
D50:	131.6 nm
D90:	195.4 nm

Stats: Mean +/- Standard Error

Mean:	143.4 +/- 2.6 nm
Mode:	119.4 +/- 4.7 nm
SD:	46.1 +/- 4.5 nm
D10:	103.0 +/- 1.8 nm
D50:	134.0 +/- 3.5 nm
D90:	195.7 +/- 7.3 nm
Concentration (Upgrade):	4.57e+08 +/- 1.67e+07 particles/ml
	37.9 +/- 2.2 particles/frame
	39.7 +/- 2.2 centres/frame

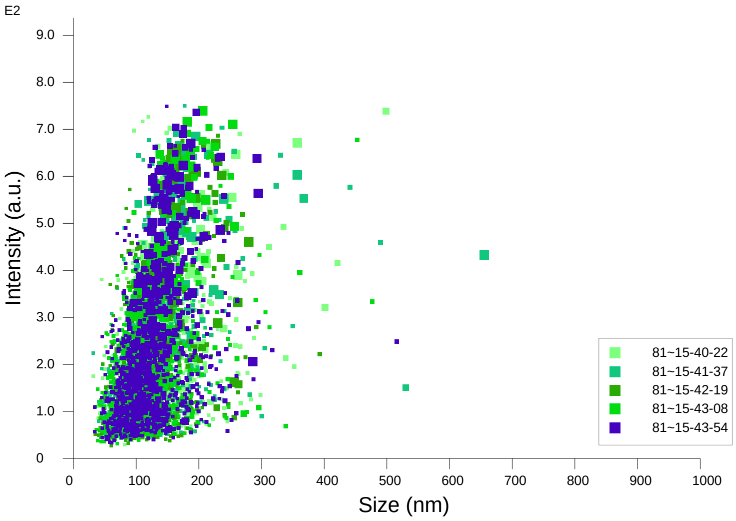

Intensity / Size graph for Experiment:
81 2023-12-07 15-40-07

Script Used: (Full Text):

SOP Standard Measurement 03-40-07PM 07Dec2023.txt
