# Supplementary material for: Neuroinflammatory responses and blood–brain barrier injury in chronic alcohol exposure: role of purinergic P2 × 7 Receptor signaling
Source: J Neuroinflammation. 2024 Sep 28;21:244. doi: 10.1186/s12974-024-03230-4 (PMC11439317; doi:10.1186/s12974-024-03230-4)
Supplement: Supplementary file 4 — Supplementary Material 4 [file 12974_2024_3230_MOESM4_ESM.pdf]

NANOSIGHT

26 2023-12-07 15-31-58

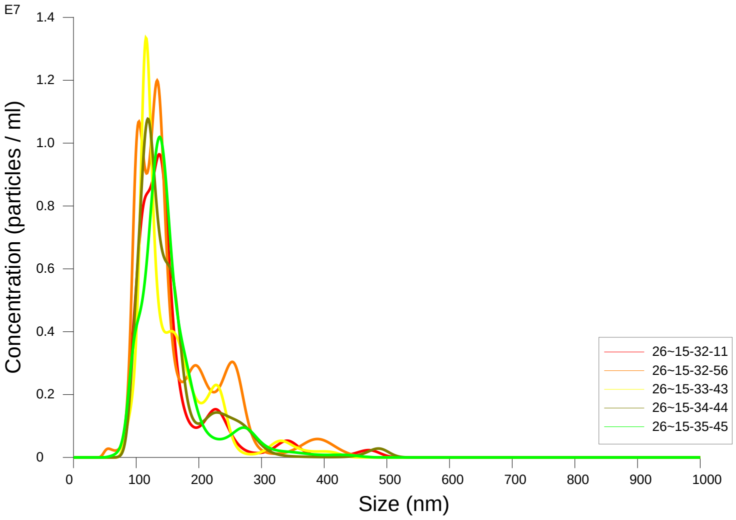

FTLA Concentration / Size graph for Experiment:
26 2023-12-07 15-31-58

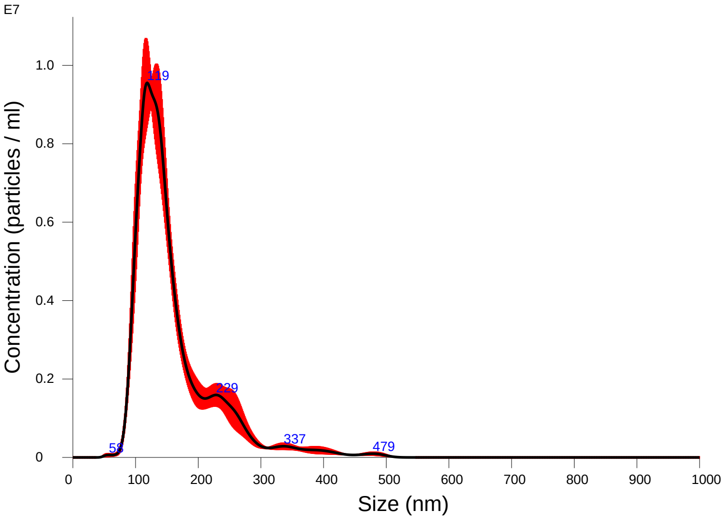

Averaged FTLA Concentration / Size for Experiment:
26 2023-12-07 15-31-58
Error bars indicate + / -1 standard error of the mean

Included Files

26 2023-12-07 15-32-11
26 2023-12-07 15-32-56
26 2023-12-07 15-33-43
26 2023-12-07 15-34-44
26 2023-12-07 15-35-45

Details

NTA Version:	NTA 3.3 Dev Build 3.3.104
Script Used:	SOP Standard Measurement 03-31-58PM 07~
Time Captured:	15:31:58 07/12/2023
Operator:	
Pre-treatment:	
Sample Name:	26
Diluent:	water
Remarks:	1:100

Capture Settings

Camera Type:	sCMOS
Laser Type:	Blue488
Camera Level:	10
Slider Shutter:	696
Slider Gain:	73
FPS	25.0
Number of Frames:	749
Temperature:	24.8 - 24.8 ^{o}C
Viscosity:	(Water) 0.892 - 0.893 cP
Dilution factor:	Dilution not recorded

Analysis Settings

Detect Threshold:	5
Blur Size:	Auto
Max Jump Distance:	Auto: 12.5 - 13.4 pix

Results

Stats: Merged Data

Mean:	138.9 nm
Mode:	118.6 nm
SD:	65.5 nm
D10:	103.2 nm
D50:	138.5 nm
D90:	245.4 nm

Stats: Mean +/- Standard Error

Mean:	138.5 +/- 1.5 nm
Mode:	120.5 +/- 4.6 nm
SD:	64.9 +/- 2.4 nm
D10:	103.6 +/- 1.0 nm
D50:	138.1 +/- 1.2 nm
D90:	241.2 +/- 4.6 nm
Concentration (Upgrade):	7.83e+08 +/- 5.40e+07 particles/ml
	69.3 +/- 5.9 particles/frame
	83.9 +/- 16.3 centres/frame

Concentration measurements may be unreliable
See summary file for more info

26 2023-12-07 15-31-58

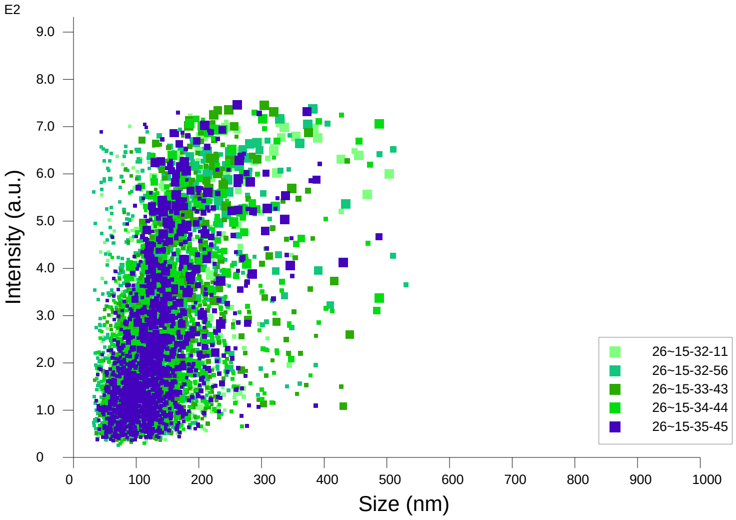

Intensity / Size graph for Experiment:
26 2023-12-07 15-31-58

Script Used: (Full Text):

SOP Standard Measurement 03-31-58PM 07Dec2023.txt
