# Supplementary material for: Neuroinflammatory responses and blood–brain barrier injury in chronic alcohol exposure: role of purinergic P2 × 7 Receptor signaling
Source: J Neuroinflammation. 2024 Sep 28;21:244. doi: 10.1186/s12974-024-03230-4 (PMC11439317; doi:10.1186/s12974-024-03230-4)
Supplement: Supplementary file 6 — Supplementary Material 6 [file 12974_2024_3230_MOESM6_ESM.pdf]

NANOSIGHT

79 2023-12-06 14-24-37

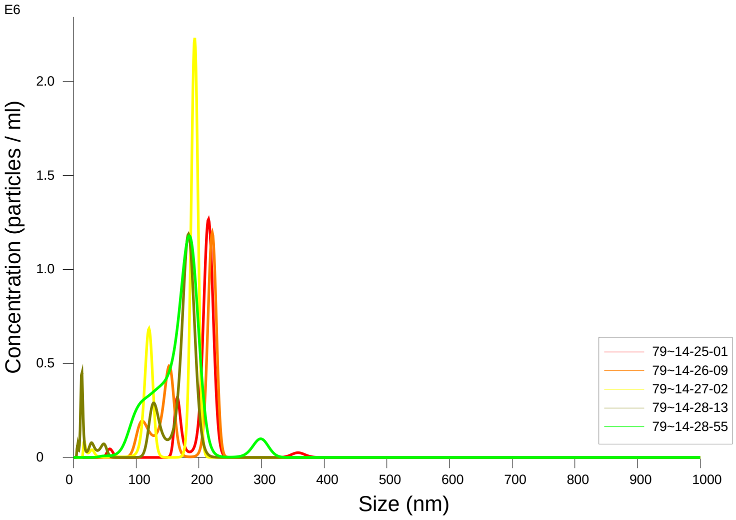

FTLA Concentration / Size graph for Experiment:
79 2023-12-06 14-24-37

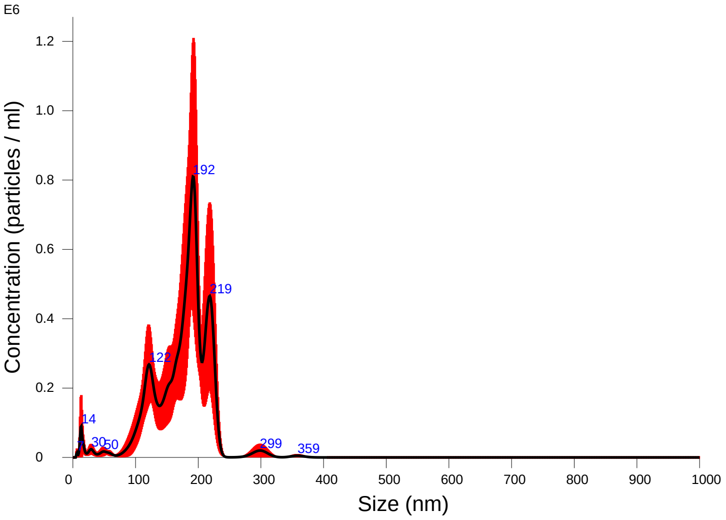

Averaged FTLA Concentration / Size for Experiment:
79 2023-12-06 14-24-37
Error bars indicate + / -1 standard error of the mean

Included Files

79 2023-12-06 14-25-01
79 2023-12-06 14-26-09
79 2023-12-06 14-27-02
79 2023-12-06 14-28-13
79 2023-12-06 14-28-55

Details

NTA Version:	NTA 3.3 Dev Build 3.3.104
Script Used:	SOP Standard Measurement 02-24-37PM 06~
Time Captured:	14:24:37 06/12/2023
Operator:	
Pre-treatment:	
Sample Name:	79
Diluent:	water
Remarks:	1:100

Capture Settings

Camera Type:	sCMOS
Laser Type:	Blue488
Camera Level:	11
Slider Shutter:	890
Slider Gain:	146
FPS	25.0
Number of Frames:	749
Temperature:	24.3 - 24.4 ^{o}C
Viscosity:	(Water) 0.902 - 0.904 cP
Dilution factor:	Dilution not recorded

Analysis Settings

Detect Threshold:	7
Blur Size:	Auto
Max Jump Distance:	Auto: 13.4 - 34.4 pix

Results

Stats: Merged Data

Mean:	175.0 nm
Mode:	191.9 nm
SD:	45.5 nm
D10:	117.1 nm
D50:	184.8 nm
D90:	219.9 nm

Stats: Mean +/- Standard Error

Mean:	178.0 +/- 8.8 nm
Mode:	199.6 +/- 7.9 nm
SD:	42.1 +/- 2.6 nm
D10:	113.3 +/- 18.3 nm
D50:	193.5 +/- 8.3 nm
D90:	209.8 +/- 6.9 nm
Concentration (Upgrade):	5.49e+08 +/- 7.17e+07 particles/ml
	4.1 +/- 0.6 particles/frame
	6.0 +/- 0.8 centres/frame

Concentration measurements may be unreliable
See summary file for more info

NANOSIGHT

79 2023-12-06 14-24-37

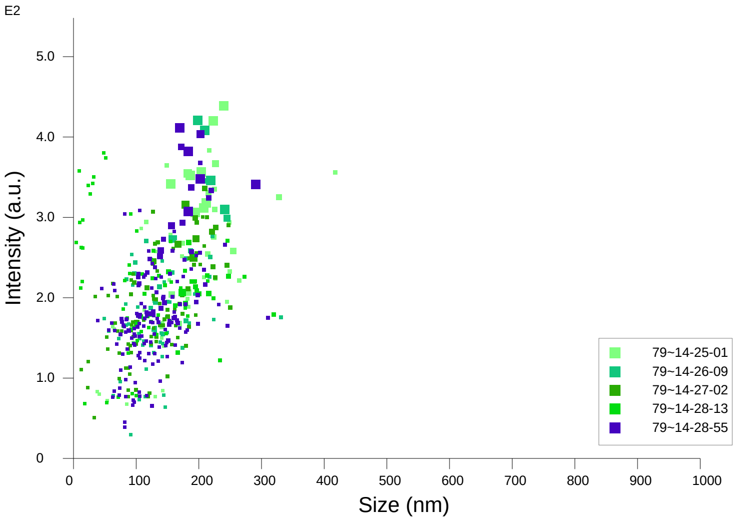

Intensity / Size graph for Experiment:
79 2023-12-06 14-24-37

Script Used: (Full Text):

SOP Standard Measurement 02-24-37PM 06Dec2023.txt
