# Supplementary material for: Neuroinflammatory responses and blood–brain barrier injury in chronic alcohol exposure: role of purinergic P2 × 7 Receptor signaling
Source: J Neuroinflammation. 2024 Sep 28;21:244. doi: 10.1186/s12974-024-03230-4 (PMC11439317; doi:10.1186/s12974-024-03230-4)
Supplement: Supplementary file 7 — Supplementary Material 7 [file 12974_2024_3230_MOESM7_ESM.pdf]

NANOSIGHT

85 2023-12-06 13-42-42

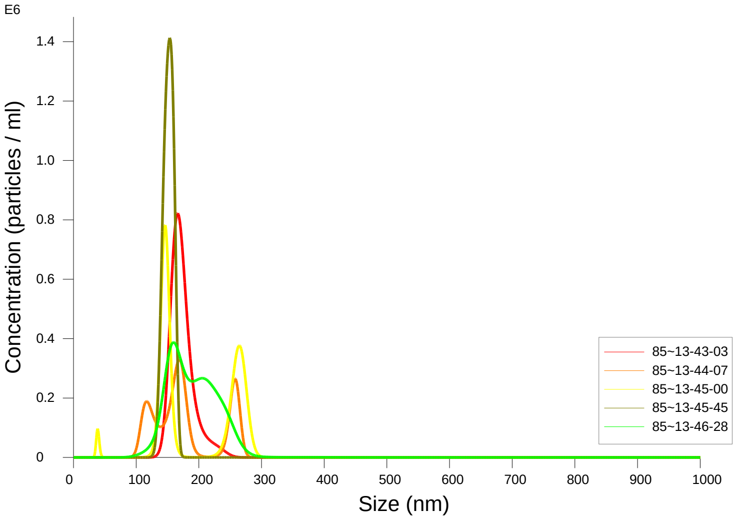

FTLA Concentration / Size graph for Experiment:
85 2023-12-06 13-42-42

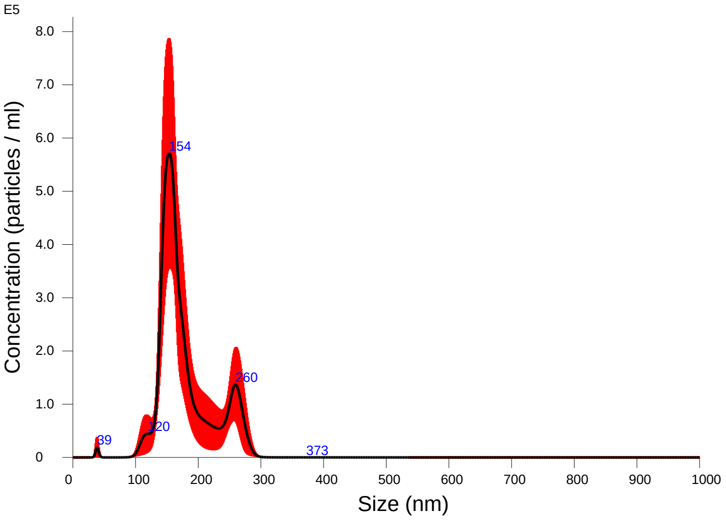

Averaged FTLA Concentration / Size for Experiment:
85 2023-12-06 13-42-42
Error bars indicate + / -1 standard error of the mean

Included Files

85 2023-12-06 13-43-03
85 2023-12-06 13-44-07
85 2023-12-06 13-45-00
85 2023-12-06 13-45-45
85 2023-12-06 13-46-28

Details

NTA Version:	NTA 3.3 Dev Build 3.3.104
Script Used:	SOP Standard Measurement 01-42-42PM 06~
Time Captured:	13:42:42 06/12/2023
Operator:	
Pre-treatment:	
Sample Name:	85
Diluent:	water
Remarks:	1:100

Capture Settings

Camera Type:	sCMOS
Laser Type:	Blue488
Camera Level:	11
Slider Shutter:	890
Slider Gain:	146
FPS	25.0
Number of Frames:	749
Temperature:	23.5 - 23.6 ^{o}C
Viscosity:	(Water) 0.917 - 0.920 cP
Dilution factor:	Dilution not recorded

Analysis Settings

Detect Threshold:	7
Blur Size:	Auto
Max Jump Distance:	Auto: 14.2 - 23.0 pix

Results

Stats: Merged Data

Mean:	177.1 nm
Mode:	153.7 nm
SD:	42.4 nm
D10:	140.3 nm
D50:	162.7 nm
D90:	253.8 nm

Stats: Mean +/- Standard Error

Mean:	177.5 +/- 7.5 nm
Mode:	158.9 +/- 4.2 nm
SD:	35.9 +/- 10.1 nm
D10:	138.9 +/- 6.1 nm
D50:	166.1 +/- 6.1 nm
D90:	226.7 +/- 20.5 nm
Concentration (Upgrade):	2.84e+07 +/- 2.15e+06 particles/ml
	2.7 +/- 0.2 particles/frame
	3.7 +/- 0.3 centres/frame

Concentration measurements may be unreliable
See summary file for more info

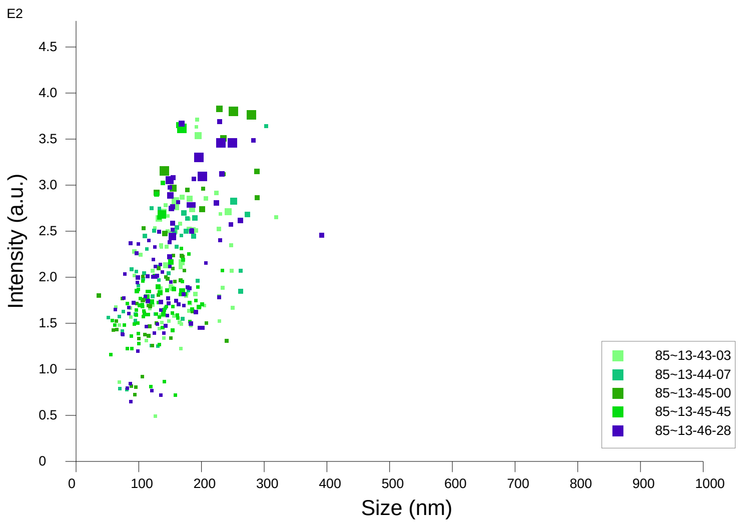

Intensity / Size graph for Experiment:
85 2023-12-06 13-42-42

Script Used: (Full Text):

SOP Standard Measurement 01-42-42PM 06Dec2023.txt
