# Supplementary material for: Neuroinflammatory responses and blood–brain barrier injury in chronic alcohol exposure: role of purinergic P2 × 7 Receptor signaling
Source: J Neuroinflammation. 2024 Sep 28;21:244. doi: 10.1186/s12974-024-03230-4 (PMC11439317; doi:10.1186/s12974-024-03230-4)
Supplement: Supplementary file 8 — Supplementary Material 8 [file 12974_2024_3230_MOESM8_ESM.pdf]

NANOSIGHT

61 2023-12-07 14-15-28

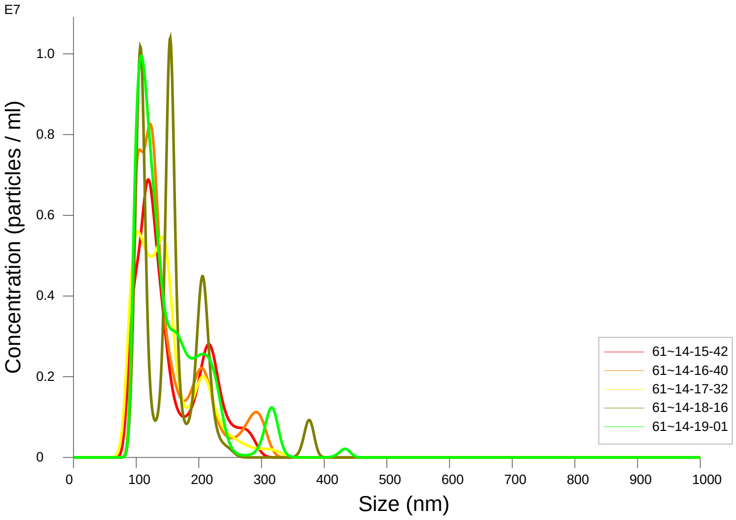

FTLA Concentration / Size graph for Experiment:
61 2023-12-07 14-15-28

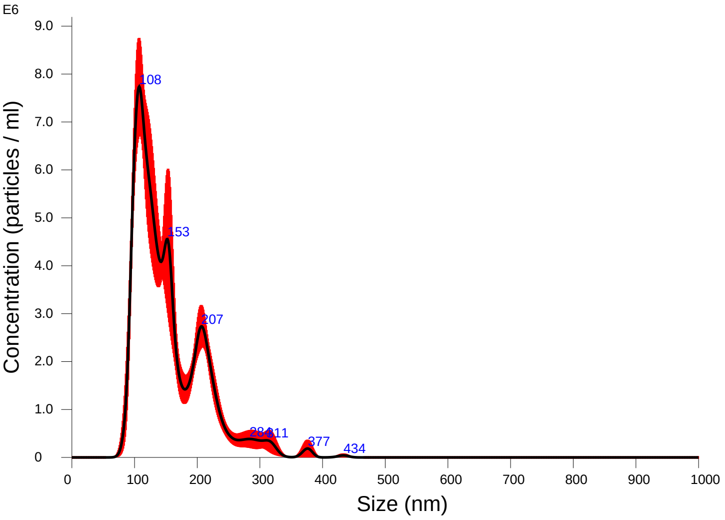

Averaged FTLA Concentration / Size for Experiment:
61 2023-12-07 14-15-28
Error bars indicate + / -1 standard error of the mean

Included Files

61 2023-12-07 14-15-42
61 2023-12-07 14-16-40
61 2023-12-07 14-17-32
61 2023-12-07 14-18-16
61 2023-12-07 14-19-01

Details

NTA Version:	NTA 3.3 Dev Build 3.3.104
Script Used:	SOP Standard Measurement 02-15-28PM 07~
Time Captured:	14:15:28 07/12/2023
Operator:	
Pre-treatment:	
Sample Name:	61
Diluent:	water
Remarks:	1:100

Capture Settings

Camera Type:	sCMOS
Laser Type:	Blue488
Camera Level:	10
Slider Shutter:	696
Slider Gain:	73
FPS	25.0
Number of Frames:	749
Temperature:	24.7 - 24.8 ^{o}C
Viscosity:	(Water) 0.893 - 0.895 cP
Dilution factor:	Dilution not recorded

Analysis Settings

Detect Threshold:	5
Blur Size:	Auto
Max Jump Distance:	Auto: 12.4 - 13.2 pix

Results

Stats: Merged Data

Mean:	153.3 nm
Mode:	107.5 nm
SD:	55.3 nm
D10:	100.2 nm
D50:	137.1 nm
D90:	224.5 nm

Stats: Mean +/- Standard Error

Mean:	153.2 +/- 1.6 nm
Mode:	121.3 +/- 9.0 nm
SD:	54.8 +/- 1.7 nm
D10:	99.8 +/- 1.2 nm
D50:	137.5 +/- 3.6 nm
D90:	225.5 +/- 4.4 nm
Concentration (Upgrade):	6.77e+08 +/- 9.69e+07 particles/ml
	49.7 +/- 2.8 particles/frame
	53.7 +/- 3.3 centres/frame

NANOSIGHT

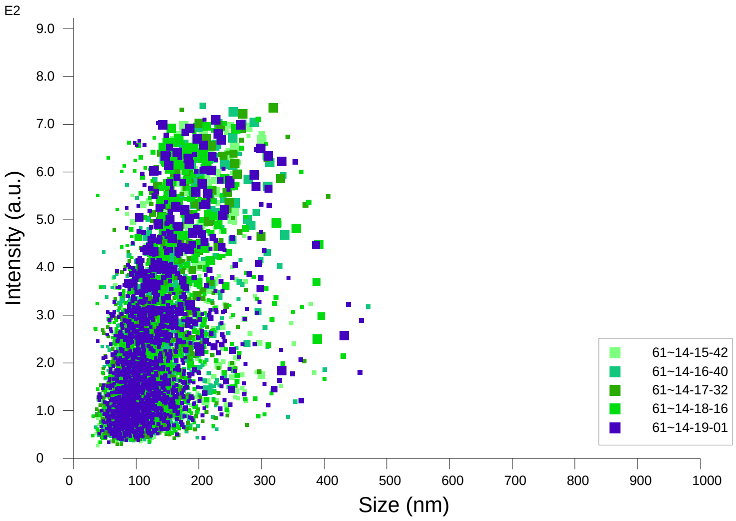

Intensity / Size graph for Experiment:
61 2023-12-07 14-15-28

Script Used: (Full Text):

SOP Standard Measurement 02-15-28PM 07Dec2023.txt
