# Supplementary material for: Neuroinflammatory responses and blood–brain barrier injury in chronic alcohol exposure: role of purinergic P2 × 7 Receptor signaling
Source: J Neuroinflammation. 2024 Sep 28;21:244. doi: 10.1186/s12974-024-03230-4 (PMC11439317; doi:10.1186/s12974-024-03230-4)
Supplement: Supplementary file 9 — Supplementary Material 9 [file 12974_2024_3230_MOESM9_ESM.pdf]

NANOSIGHT

65 2023-12-07 14-45-33

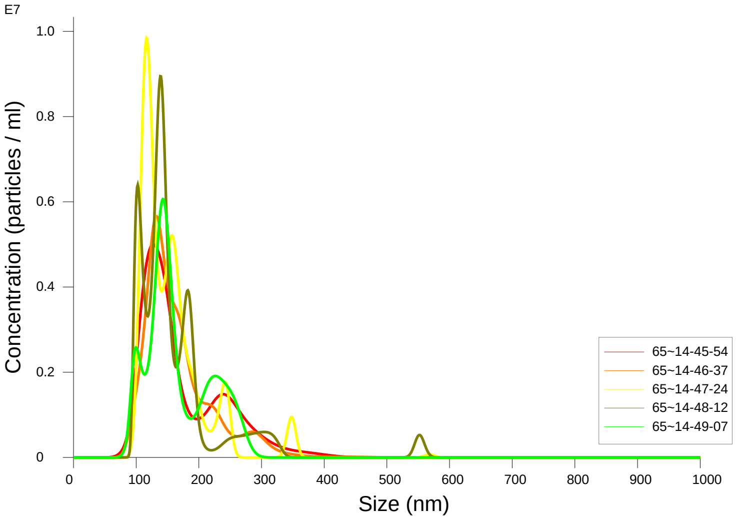

FTLA Concentration / Size graph for Experiment:
65 2023-12-07 14-45-33

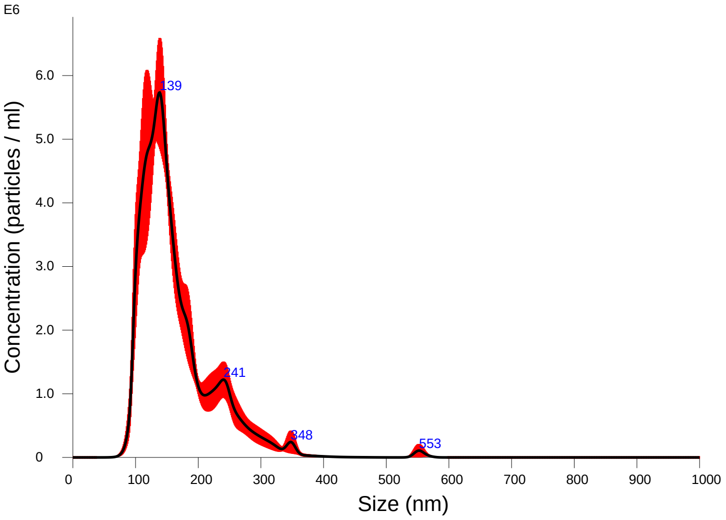

Averaged FTLA Concentration / Size for Experiment:
65 2023-12-07 14-45-33
Error bars indicate + / -1 standard error of the mean

Included Files

65 2023-12-07 14-45-54
65 2023-12-07 14-46-37
65 2023-12-07 14-47-24
65 2023-12-07 14-48-12
65 2023-12-07 14-49-07

Details

NTA Version:	NTA 3.3 Dev Build 3.3.104
Script Used:	SOP Standard Measurement 02-45-33PM 07~
Time Captured:	14:45:33 07/12/2023
Operator:	
Pre-treatment:	
Sample Name:	65
Diluent:	water
Remarks:	1:100

Capture Settings

Camera Type:	sCMOS
Laser Type:	Blue488
Camera Level:	10
Slider Shutter:	696
Slider Gain:	73
FPS	25.0
Number of Frames:	749
Temperature:	24.9 - 24.9 ^{o}C
Viscosity:	(Water) 0.890 - 0.891 cP
Dilution factor:	Dilution not recorded

Analysis Settings

Detect Threshold:	5
Blur Size:	Auto
Max Jump Distance:	Auto: 12.1 - 12.9 pix

Results

Stats: Merged Data

Mean:	163.9 nm
Mode:	138.2 nm
SD:	61.3 nm
D10:	107.9 nm
D50:	146.2 nm
D90:	245.8 nm

Stats: Mean +/- Standard Error

Mean:	164.3 +/- 2.6 nm
Mode:	131.6 +/- 4.6 nm
SD:	60.0 +/- 4.5 nm
D10:	108.2 +/- 1.6 nm
D50:	146.6 +/- 2.1 nm
D90:	247.0 +/- 6.7 nm
Concentration (Upgrade):	8.85e+08 +/- 2.42e+08 particles/ml
	46.9 +/- 1.1 particles/frame
	49.7 +/- 1.2 centres/frame

NANOSIGHT

65 2023-12-07 14-45-33

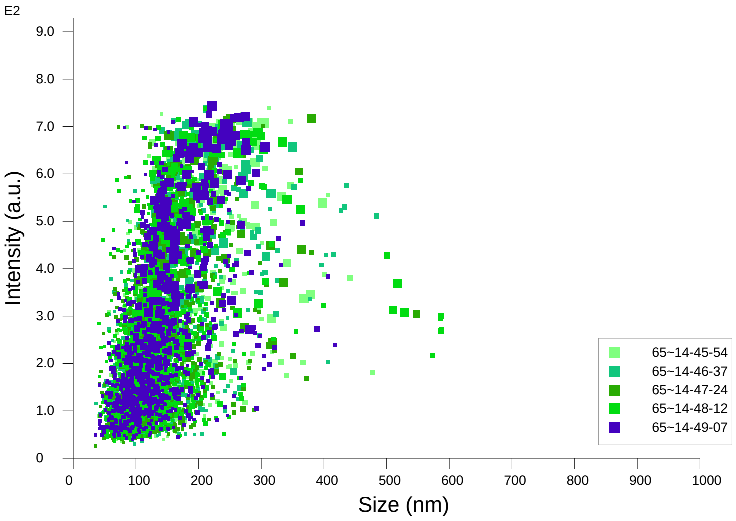

Intensity / Size graph for Experiment:
65 2023-12-07 14-45-33

Script Used: (Full Text):

SOP Standard Measurement 02-45-33PM 07Dec2023.txt
