# Supplementary material for: Neuroinflammatory responses and blood–brain barrier injury in chronic alcohol exposure: role of purinergic P2 × 7 Receptor signaling
Source: J Neuroinflammation. 2024 Sep 28;21:244. doi: 10.1186/s12974-024-03230-4 (PMC11439317; doi:10.1186/s12974-024-03230-4)
Supplement: Supplementary file 10 — Supplementary Material 10 [file 12974_2024_3230_MOESM10_ESM.pdf]

NANOSIGHT

35 2023-12-07 15-53-42

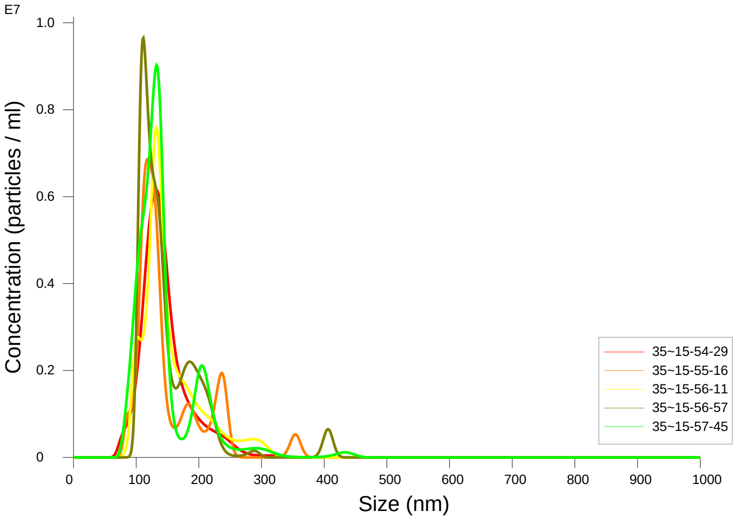

FTLA Concentration / Size graph for Experiment:
35 2023-12-07 15-53-42

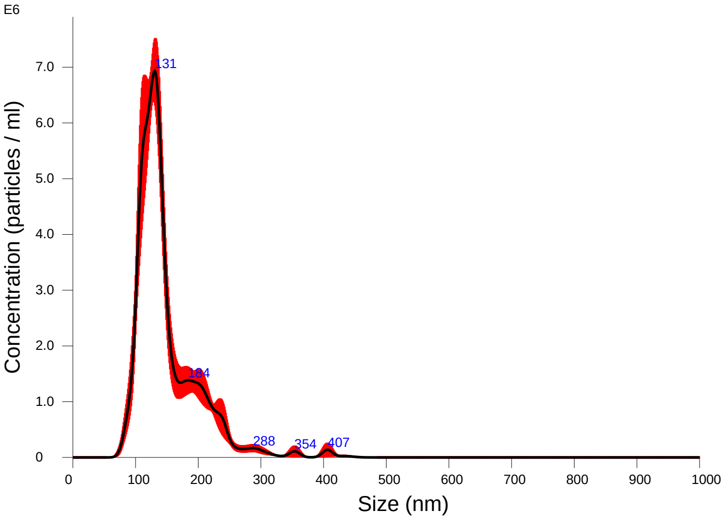

Averaged FTLA Concentration / Size for Experiment:
35 2023-12-07 15-53-42
Error bars indicate + / -1 standard error of the mean

Included Files

35 2023-12-07 15-54-29
35 2023-12-07 15-55-16
35 2023-12-07 15-56-11
35 2023-12-07 15-56-57
35 2023-12-07 15-57-45

Details

NTA Version:	NTA 3.3 Dev Build 3.3.104
Script Used:	SOP Standard Measurement 03-53-42PM 07~
Time Captured:	15:53:42 07/12/2023
Operator:	
Pre-treatment:	
Sample Name:	35
Diluent:	water
Remarks:	1:100

Capture Settings

Camera Type:	sCMOS
Laser Type:	Blue488
Camera Level:	10
Slider Shutter:	696
Slider Gain:	73
FPS	25.0
Number of Frames:	749
Temperature:	24.8 - 24.8 ^{o}C
Viscosity:	(Water) 0.892 - 0.893 cP
Dilution factor:	Dilution not recorded

Analysis Settings

Detect Threshold:	5
Blur Size:	Auto
Max Jump Distance:	Auto: 12.7 - 14.2 pix

Results

Stats: Merged Data

Mean:	138.9 nm
Mode:	130.4 nm
SD:	50.9 nm
D10:	105.0 nm
D50:	133.8 nm
D90:	214.8 nm

Stats: Mean +/- Standard Error

Mean:	139.0 +/- 1.7 nm
Mode:	125.2 +/- 4.4 nm
SD:	50.2 +/- 3.2 nm
D10:	105.0 +/- 1.2 nm
D50:	133.8 +/- 1.9 nm
D90:	215.0 +/- 6.1 nm
Concentration (Upgrade):	4.36e+08 +/- 2.42e+07 particles/ml
	33.9 +/- 1.9 particles/frame
	35.6 +/- 1.8 centres/frame

NANOSIGHT

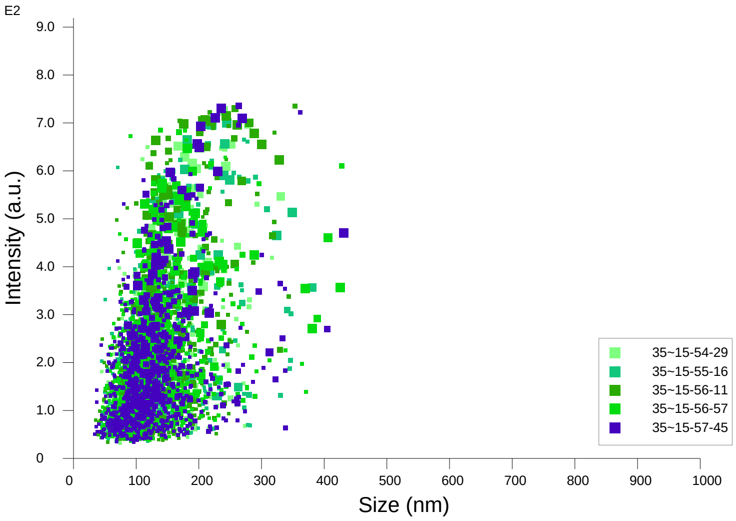

Intensity / Size graph for Experiment:
35 2023-12-07 15-53-42

Script Used: (Full Text):

SOP Standard Measurement 03-53-42PM 07Dec2023.txt
