# Supplementary material for: Neuroinflammatory responses and blood–brain barrier injury in chronic alcohol exposure: role of purinergic P2 × 7 Receptor signaling
Source: J Neuroinflammation. 2024 Sep 28;21:244. doi: 10.1186/s12974-024-03230-4 (PMC11439317; doi:10.1186/s12974-024-03230-4)
Supplement: Supplementary file 11 — Supplementary Material 11 [file 12974_2024_3230_MOESM11_ESM.pdf]

NANOSIGHT

32 2023-12-07 16-09-19

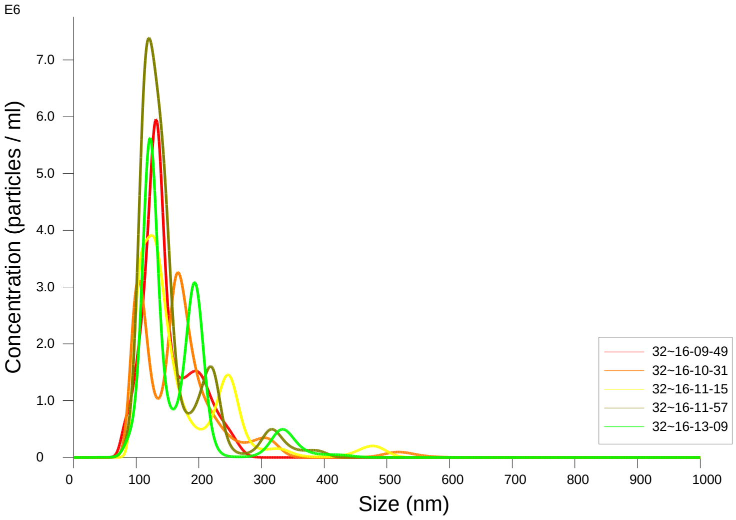

FTLA Concentration / Size graph for Experiment:
32 2023-12-07 16-09-19

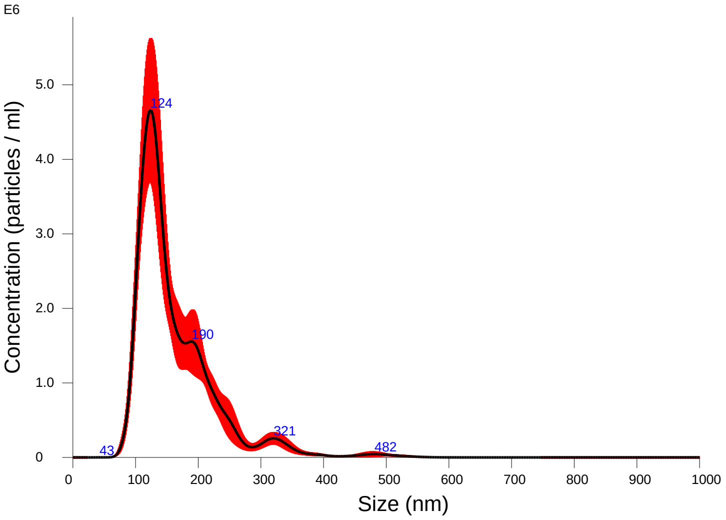

Averaged FTLA Concentration / Size for Experiment:
32 2023-12-07 16-09-19
Error bars indicate + / -1 standard error of the mean

Included Files

32 2023-12-07 16-09-49
32 2023-12-07 16-10-31
32 2023-12-07 16-11-15
32 2023-12-07 16-11-57
32 2023-12-07 16-13-09

Details

NTA Version:	NTA 3.3 Dev Build 3.3.104
Script Used:	SOP Standard Measurement 04-09-19PM 07~
Time Captured:	16:09:19 07/12/2023
Operator:	
Pre-treatment:	
Sample Name:	32
Diluent:	water
Remarks:	1:100

Capture Settings

Camera Type:	sCMOS
Laser Type:	Blue488
Camera Level:	10
Slider Shutter:	696
Slider Gain:	73
FPS	25.0
Number of Frames:	749
Temperature:	24.7 ^{o}C
Viscosity:	(Water) 0.894 - 0.895 cP
Dilution factor:	Dilution not recorded

Analysis Settings

Detect Threshold:	5
Blur Size:	Auto
Max Jump Distance:	Auto: 12.3 - 13.3 pix

Results

Stats: Merged Data

Mean:	162.0 nm
Mode:	123.9 nm
SD:	65.2 nm
D10:	105.5 nm
D50:	141.1 nm
D90:	237.6 nm

Stats: Mean +/- Standard Error

Mean:	162.8 +/- 4.4 nm
Mode:	133.1 +/- 8.6 nm
SD:	63.9 +/- 6.8 nm
D10:	105.5 +/- 1.7 nm
D50:	143.9 +/- 4.9 nm
D90:	234.2 +/- 9.2 nm
Concentration (Upgrade):	3.68e+08 +/- 2.93e+07 particles/ml
	30.2 +/- 1.1 particles/frame
	31.7 +/- 1.1 centres/frame

NANOSIGHT

32 2023-12-07 16-09-19

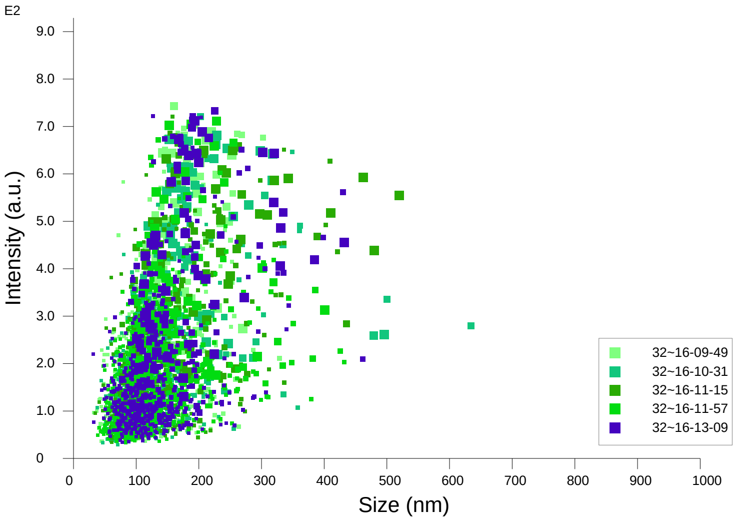

Intensity / Size graph for Experiment:
32 2023-12-07 16-09-19

Script Used: (Full Text):

SOP Standard Measurement 04-09-19PM 07Dec2023.txt
