# Supplementary material for: Neuroinflammatory responses and blood–brain barrier injury in chronic alcohol exposure: role of purinergic P2 × 7 Receptor signaling
Source: J Neuroinflammation. 2024 Sep 28;21:244. doi: 10.1186/s12974-024-03230-4 (PMC11439317; doi:10.1186/s12974-024-03230-4)
Supplement: Supplementary file 12 — Supplementary Material 12 [file 12974_2024_3230_MOESM12_ESM.pdf]

NANOSIGHT

83 2023-12-07 16-01-28

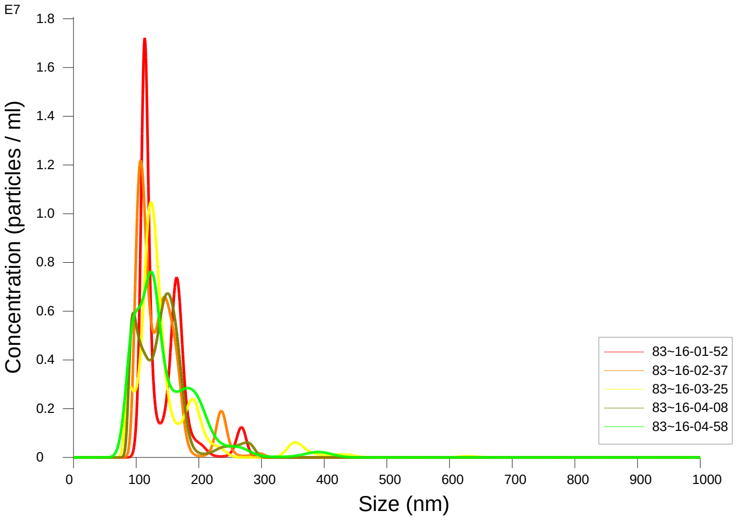

FTLA Concentration / Size graph for Experiment:
83 2023-12-07 16-01-28

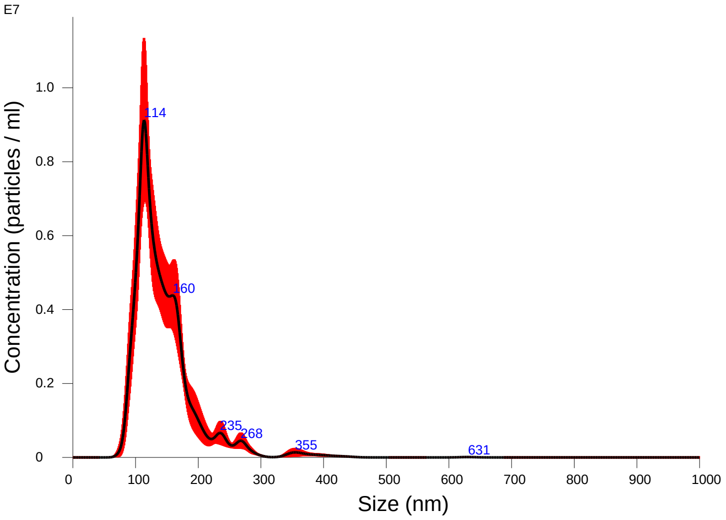

Averaged FTLA Concentration / Size for Experiment:
83 2023-12-07 16-01-28
Error bars indicate + / -1 standard error of the mean

Included Files

83 2023-12-07 16-01-52
83 2023-12-07 16-02-37
83 2023-12-07 16-03-25
83 2023-12-07 16-04-08
83 2023-12-07 16-04-58

Details

NTA Version:	NTA 3.3 Dev Build 3.3.104
Script Used:	SOP Standard Measurement 04-01-28PM 07~
Time Captured:	16:01:28 07/12/2023
Operator:	
Pre-treatment:	
Sample Name:	83
Diluent:	water
Remarks:	1:100

Capture Settings

Camera Type:	sCMOS
Laser Type:	Blue488
Camera Level:	10
Slider Shutter:	696
Slider Gain:	73
FPS	25.0
Number of Frames:	749
Temperature:	24.7 °C
Viscosity:	(Water) 0.893 - 0.894 cP
Dilution factor:	Dilution not recorded

Analysis Settings

Detect Threshold:	5
Blur Size:	Auto
Max Jump Distance:	Auto: 13.1 - 13.7 pix

Results

Stats: Merged Data

Mean:	144.3 nm
Mode:	113.7 nm
SD:	50.5 nm
D10:	100.3 nm
D50:	131.6 nm
D90:	197.5 nm

Stats: Mean +/- Standard Error

Mean:	144.3 +/- 2.1 nm
Mode:	123.7 +/- 7.3 nm
SD:	48.7 +/- 5.9 nm
D10:	100.2 +/- 2.6 nm
D50:	131.3 +/- 2.4 nm
D90:	190.5 +/- 7.2 nm
Concentration (Upgrade):	5.62e+08 +/- 2.29e+07 particles/ml
	39.1 +/- 0.9 particles/frame
	40.8 +/- 0.9 centres/frame

NANOSIGHT

83 2023-12-07 16-01-28

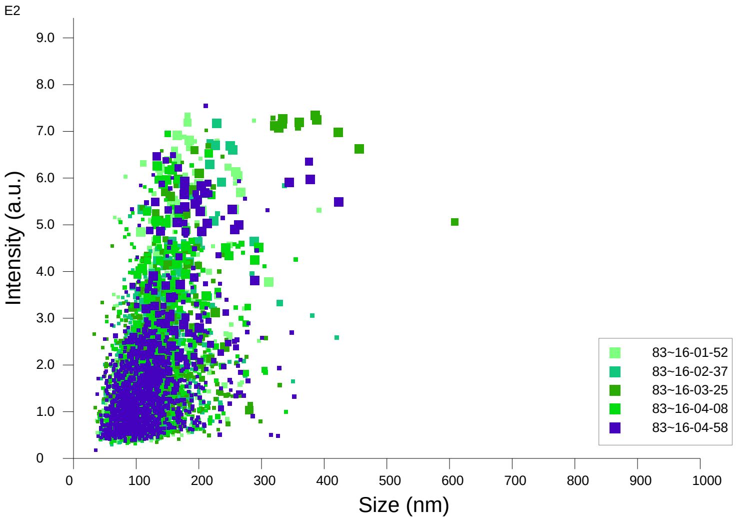

Intensity / Size graph for Experiment:
83 2023-12-07 16-01-28

Script Used: (Full Text):

SOP Standard Measurement 04-01-28PM 07Dec2023.txt
